# Induction of New Lactam Derivatives From the Endophytic Fungus *Aplosporella javeedii* Through an OSMAC Approach

**DOI:** 10.3389/fmicb.2020.600983

**Published:** 2020-11-04

**Authors:** Ying Gao, Fabian Stuhldreier, Laura Schmitt, Sebastian Wesselborg, Zhiyong Guo, Kun Zou, Attila Mándi, Tibor Kurtán, Zhen Liu, Peter Proksch

**Affiliations:** ^1^Institute of Pharmaceutical Biology and Biotechnology, Heinrich Heine University Düsseldorf, Düsseldorf, Germany; ^2^Institute of Molecular Medicine I, Medical Faculty, Heinrich Heine University Düsseldorf, Düsseldorf, Germany; ^3^Hubei Key Laboratory of Natural Products Research and Development, College of Biological and Pharmaceutical Sciences, China Three Gorges University, Yichang, China; ^4^Department of Organic Chemistry, University of Debrecen, Debrecen, Hungary

**Keywords:** *Aplosporella javeedii*, lactam derivatives, OSMAC approach, DFT-NMR, TDDFT-ECD, OR calculations, apoptosis

## Abstract

Fermentation of the endophytic fungus *Aplosporella javeedii* on solid rice medium in presence of either 3.5% NaNO_3_ or 3.5% monosodium glutamate caused a significant change of the fungal metabolite pattern compared to fungal controls grown only on rice. Chemical investigation of the former fungal extracts yielded 11 new lactam derivatives, aplosporellins A–K (**2**–**12**), in addition to the known compound, pramanicin A (**1**). All of these compounds were not detected when the fungus was grown on rice medium without these activators thereby indicating the power of this OSMAC approach. The structures of the new compounds were elucidated by one- and two- dimensional NMR spectroscopy, DFT-NMR calculations and by mass spectrometry as well as by comparison with the literature whereas the absolute configuration of the lactam core was determined by TDDFT-ECD and OR calculations. Pramanicin A (**1**) showed strong cytotoxicity against human lymphoma (Ramos) and leukemia (Jurkat J16) cells with IC_50_ values of 4.7 and 4.4 μM, respectively. Mechanistic studies indicated that **1** activates caspase-3 and induces apoptotic cell death.

## Introduction

Endophytic fungi have been proven to be important sources for bioprospecting for new pharmaceutical lead compounds (Frank et al., 2015; Ancheeva et al., 2018; Bohler et al., 2018; Rehberg et al., 2018). However, conventional screening of endophytes that had been cultivated under standard laboratory conditions often fails to reveal the full biosynthetic potential of fungi and leads to re-isolation of already known metabolites. Strategies to activate silent biosynthetic gene clusters that are not expressed using conventional fermentation methods include co-cultivation of fungi with bacteria or the so called OSMAC (One Strain Many Compounds) approach (Daletos et al., 2017). The OSMAC approach makes use of altering cultivation parameters such as medium composition (carbon/nitrogen ratio, salinity, metal ions), physical parameters (temperature, pH, oxygen condition), or addition of enzyme inhibitors/inducers and biosynthetic precursors in order to activate silent biosynthetic gene clusters and to expand the metabolite pattern produced by endophytes (Bode et al., 2002; Pan et al., 2019). Recent successful examples of OSMAC application from our own group include: addition of 2% tryptophan to rice medium which led to the accumulation of a new strongly cytotoxic bismacrolactone by the endophytic fungus *Trichocladium* sp. (Tran-Cong et al., 2019), addition of a mixture of salts (MgSO_4_, NaNO_3_, and NaCl) to solid Czapek medium which induced accumulation of nine new secondary metabolites by the endophytic fungus *Bulgaria inquinans* (Ariantari et al., 2019), and the accumulation of new brominated tyrosine-derived alkaloids by the soil fungus *Gymnascella dankaliensis* caused by addition of NaBr to solid rice medium (Wang et al., 2016).

**FIGURE 1:**
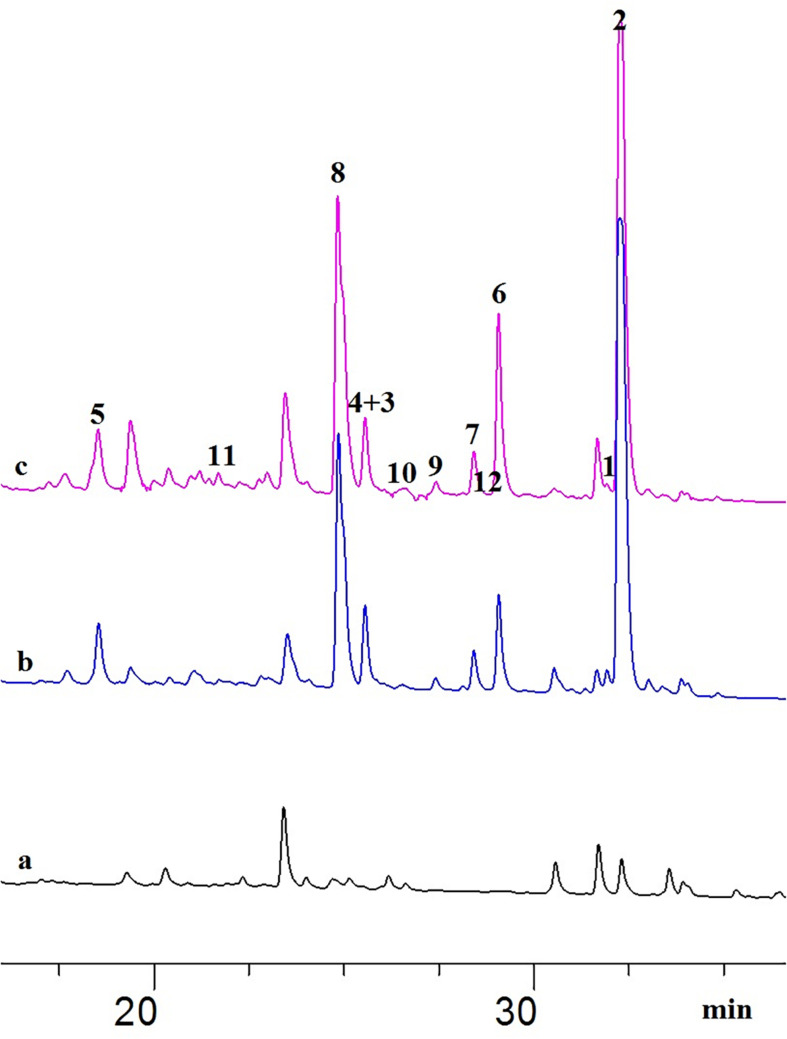
HPLC chromatograms of the EtOAc extracts from OSMAC experiments detected at 235 nm: **(A)** *A. javeedii* control grown on solid rice medium; **(B)** *A. javeedii* cultured on solid rice medium with 3.5% NaNO_3_; **(C)** *A. javeedii* cultured on solid rice medium with 3.5% monosodium glutamate.

**FIGURE 2:**
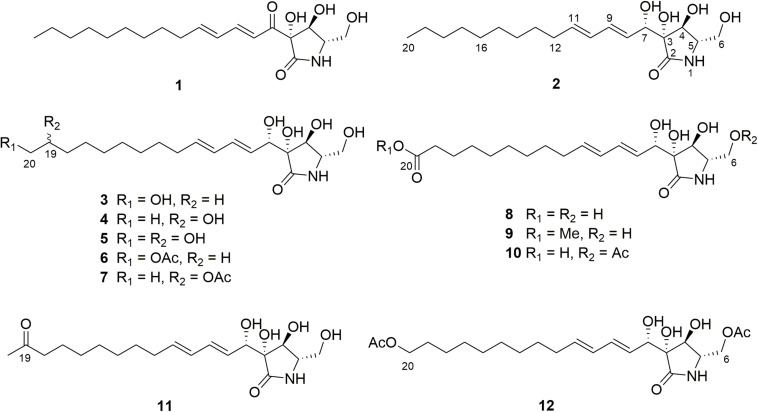
Structures of compounds isolated from *A. javeedii*.

As a part of our ongoing studies on fungal endophytes, we investigated the endophytic fungus *Aplosporella javeedii* derived from *Orychophragmus violaceus* (L.) O. E. Schul (Brassicaceae). *O. violaceus* is used in the Traditional Medicine of China for dissipating swelling and for treating unknown pyrogenic infections (Medicinal Plant Images Database, 2007). Recent studies have found that the plant also shows hepatoprotective effects (Huo et al., 2017). Previous chemical investigations of the fungus *A. javeedii* grown on solid rice medium resulted in the isolation of six new antifungal polyketides, five sesterterpenes including two new compounds, as well as a new macrolide, with some of the metabolites exhibiting cytotoxic and antimicrobial activities (Gao et al., 2020a, b). Due to the pronounced chemical diversity of natural products obtained from this fungus, we have now conducted an OSMAC study which involved the addition of different salts including 3.5% NaBr, 3.5% NaCl, 3.5% NaF, 3.5% KCl, 3.5% NH_4_Cl, 3.5% (NH_4_)_2_SO_4_, 3.5% C_5_H_8_NNaO_4_⋅H_2_O (monosodium glutamate), 3.5% NaNO_3_, 3.5% Na_2_HPO_4_, 3.5% K_2_HPO_4_⋅3H_2_O, 3.5% KH_2_PO_4_, 3.5% FeSO_4_, 3.5% ZnSO_4_, or 3.5% MgSO_4_ to solid rice medium. The selection of most of these salts was based on previous studies which indicated their usefulness for activation of non-transcribed biosynthetic gene clusters (Hammerschmidt et al., 2015; Wang et al., 2016, 2018; Ariantari et al., 2019). The most striking effects with regard to an alteration of the fungal metabolite pattern, however, were detected following addition of either 3.5% NaNO_3_ or of 3.5% monosodium glutamate to solid rice medium compared to fungal control cultures lacking either of these activators (Figure 1). Chemical investigation of fungal extracts obtained from fermentation of *A. javeedii* in presence of either 3.5% NaNO_3_ or of 3.5% monosodium glutamate led to the isolation of 11 new lactam derivatives, aplosporellins A–K (**2**–**12**), in addition to the known compound, pramanicin A (**1**) (Figure 2), all of which were not detected when the fungus was grown on rice medium without these activators. Herein we report the structure elucidation of the new metabolites and the pro-apoptotic activity of pramanicin A (**1**).

## Materials and Methods

### General Experimental Procedures

A Perkin-Elmer-241 MC polarimeter was used to measure optical rotations. ECD spectra were recorded on a J-810 spectropolarimeter. One- and two-dimensional NMR spectra were recorded on a Bruker ARX 600 spectrometer. Mass spectra (ESI) were recorded with a Finnigan LCQ Deca mass spectrometer. A UHR-QTOF maxis 4G mass spectrometer (Bruker Daltonics) was used to record HRESIMS data. A Dionex UltiMate-3400SD system with a LPG-3400SD pump and a photodiode array detector (DAD 3000RS) as well as a separation column (Eurosphere-10 C_18_, 125 × 4 mm, Knauer) were used for HPLC analysis. Detection wave lengths were set at 235, 254, 280, and 340 nm. Semi-preparative HPLC analysis was performed with a Merck Hitachi Chromaster HPLC system (UV detector L7400; pump L7100; column Eurosphere-100 C_18_, 300 × 8 mm, Knauer; flow rate at 5 mL/min). Silica gel 60 M (0.04–0.063 mm, Macherey-Nagel) or Sephadex LH-20 were used for column chromatography. TLC plates precoated with silica gel F_254_ (Merck) were used to monitor isolation fractions. Distilled and spectral grade solvents were used for column chromatography and spectroscopic measurements, respectively.

### Fungal Material and Fermentation

The fungus *A. javeedii* (ID code ZGB-B) was isolated from fresh, healthy stems of *Orychophragmus violaceus* (L.) O. E. Schul (*Brassicaceae*), collected in April 2018 in Beijing, China. Fungal identification was carried out according to a standard protocol as described previously (Kjer et al., 2010). The GenBank accession number is MN720704. The fungal strain is kept in the Institute of Pharmaceutical Biology and Biotechnology, Heinrich Heine University, Duesseldorf, Germany.

The fungus was cultivated in two 1 L Erlenmeyer flasks, of which each was filled with solid rice medium containing 100 g rice and 110 mL demineralized water. After autoclaving at 121°C for 20 min and cooling down to room temperature, the fungal strain that was preserved on the ager plats for a week was cut into pieces and added in each flask under sterile condition. The fermentation was maintained under static conditions at room temperature until the rice medium was completely overgrown by the fungus which lasted around 20 days (control cultivation). OSMAC cultivations were carried out following the same procedure by growing the fungus on solid rice medium containing 3.5% NaBr, 3.5% NaCl, 3.5% NaF, 3.5% KCl, 3.5% NH_4_Cl, 3.5% (NH_4_)_2_SO_4_, 3.5% C_5_H_8_NNaO_4_⋅H_2_O (monosodium glutamate), 3.5% NaNO_3_, 3.5% Na_2_HPO_4_, 3.5% K_2_HPO_4_⋅3H_2_O, 3.5% KH_2_PO_4_, 3.5% FeSO_4_, 3.5% ZnSO_4_, or 3.5% MgSO_4_. Two flasks were used for each experiment and each flask contained 100 g rice, 110 mL demineralized water and 3.5 g salts. The usefulness of 3.5% salts in the OSMAC approach has been proved by previous experiments with other fungi (Hammerschmidt et al., 2015; Wang et al., 2016, 2018). Based on the chromatographic profiles obtained from the extractions of these fermentations, fungal cultivations with striking changes of metabolite patterns were selected for further investigation.

### Extraction and Isolation

The fungal culture grown on solid rice medium with addition of 3.5% NaNO_3_ or 3.5% monosodium glutamate was extracted with 800 mL EtOAc followed by evaporation to dryness to afford the crude extract. The obtained brown extracts from the 3.5% NaNO_3_ and 3.5% monosodium glutamate cultures were 2.3 and 2.8 g, respectively. The two crude extracts were subjected to a silica gel vacuum liquid chromatography (VLC) column, and eluted with 100% *n*-hexane, *n*-hexane-EtOAc (9:1), *n*-hexane-EtOAc (1:1), 100% EtOAc, CH_2_Cl_2_-MeOH (1:1), and 100% MeOH, respectively, which resulted in 6 fractions (V1 to V6) for each extract.

From the 3.5% NaNO_3_ culture extract, fraction V5 (0.55 g) was subjected to a Sephadex LH-20 column using 100% MeOH as eluent to give five subfractions (V5-S1 to V5-S5). Subfraction V5-S2 was then submitted to a RP-18 (40–63 μm) vacuum liquid chromatography column and eluted with 5–100% aqueous MeOH to yield 7 subfractions (V5-S2-RP1 to V5-S2-RP7). Subfraction V5-S2-RP4 was purified by semi-preparative HPLC using a mixture of MeCN and H_2_O (10:90) containing 0.1% HCOOH to give **4** (4.5 mg), **8** (8.2 mg), and **11** (4.3 mg). Subfraction V5-S2-RP5 was purified by semi-preparative HPLC using a gradient of MeCN and H_2_O (15:85 to 50:50) containing 0.1% HCOOH to give **2** (23.5 mg), **3** (5.1 mg), **6** (6.0 mg), and **7** (5.9 mg).

From the 3.5% monosodium glutamate culture extract, fraction V4 (0.45 g) was subjected to a Sephadex LH-20 column using CH_2_Cl_2_-MeOH (1:1) as eluent to obtain three subfractions (V4-S1 to V4-S3). Subfraction V4-S2 was then submitted to RP-18 (40–63 μm) vacuum liquid chromatography column and eluted with 10–100% aqueous MeOH to yield 10 subfractions (V4-S2-RP1 to V4-S2-RP10). Subfraction V4-S2-RP5 was purified by semi-preparative HPLC using MeOH-0.1% HCOOH in H_2_O (50:50 to 72:28) to give **10** (2.3 mg). Subfraction V4-S2-RP7 was purified by semi-preparative HPLC using MeOH-0.1% HCOOH in H_2_O (65:35 to 86:14) to give **1** (25 mg) and **12** (1.4 mg). Fraction V5 (0.68 g) was subjected to a Sephadex LH-20 column using 100% MeOH as eluent to obtain eight subfractions (V5-S1 to V5-S8). Subfraction V5-S4 was submitted to a RP-18 (40–63 μm) vacuum liquid chromatography column and eluted with 10–100% aqueous MeOH to yield 10 subfractions (V5-S4-RP1 to V5-S4-RP10). Subfraction V5-S4-RP4 was purified by semi-preparative HPLC using MeOH-0.1% HCOOH in H_2_O (50:50 to 70:30) to give **5** (2.5 mg). Subfraction V5-S4-RP6 was purified by semi-preparative HPLC using MeOH-0.1% HCOOH in H_2_O (60:40) to give **9** (3.0 mg).

Pramanicin A (**1)**: white solid; [α]^20^_D_ -121 (*c* 0.1, MeOH).

Aplosporellin A (**2**): Colorless oil; [α]^20^_D_ -21 (*c* 0.1, MeOH); UV (MeOH) λ_max_ 233 nm; ECD λ [nm] (ϕ): 239 (−1.54), 219 (1.06), 197 (−8.99); ^1^H and ^13^C NMR data, see Table 1; HRESIMS [M + Na]^+^ *m*/*z* 378.2252 (calcd for C_19_H_33_NNaO_5_ 378.2251) (Supplementary Figures S1–S13).

**TABLE 1:** ^1^H and ^13^C NMR data of compounds **2 and 3**.

No.	2*^*a*^*		2*^*b*^*		3*^*b*^*	
	**δ_*C*_, type**	**δ_*H*_ (*J* in Hz)**	**δ_*C*_, type**	**δ_*H*_ (*J* in Hz)**	**δ_*C*_, type**	**δ_*H*_ (*J* in Hz)**
1	NH	7.69, br s				
2	174.4, C		177.3, C		177.4, C	
3	78.8, C		80.8, C		80.8, C	
4	76.1, CH	3.89, t (5.9)	77.9, CH	4.12, d (6.6)	78.0, CH	4.12, d (6.5)
5	59.5, CH	3.16, ddd (5.9, 5.5, 3.5)	61.2, CH	3.38, ddd (6.6, 5.8, 3.2)	61.2, CH	3.38, ddd (6.5, 5.8, 3.3)
6	61.6, CH_2_	3.54, ddd (11.1, 5.0, 3.5) 3.30, ddd (11.1, 5.5, 5.0)	62.9, CH_2_	3.76, dd (11.6, 3.2) 3.52, dd (11.6, 5.8)	62.9, CH_2_	3.76, dd (11.6, 3.3) 3.52, dd (11.6, 5.8)
7	73.0, CH	4.22, dd (6.5, 5.9)	74.5, CH	4.43, d (7.1)	74.6, CH	4.43, d (7.0)
8	130.8, CH	5.82, dd (15.4, 6.5)	129.6, CH	5.83, dd (15.4, 7.1)	129.6, CH	5.82, dd (15.4, 7.0)
9	131.0, CH	6.14, dd (15.4, 10.6)	134.6, CH	6.32, dd (15.4, 10.4)	134.6, CH	6.32, dd (15.4, 10.5)
10	130.2, CH	6.00, dd (15.1, 10.6)	131.2, CH	6.07, dd (15.0, 10.4)	131.2, CH	6.07, dd (15.0, 10.5)
11	133.6, CH	5.62, dt (15.1, 7.3)	136.3, CH	5.70, dt (15.0, 7.3)	136.3, CH	5.70, dt (15.0, 7.3)
12	32.0, CH_2_	2.04, q (7.3)	33.7, CH_2_	2.08, q (7.3)	33.6, CH_2_	2.08, q (7.3)
13	28.8, CH_2_	1.34, m	30.5, CH_2_	1.39, m	30.4, CH_2_	1.39, m
14	29.0, CH_2_	1.24, m	30.7, CH_2_	1.29, m	30.7, CH_2_	1.31, m
15	28.9, CH_2_	1.24, m	30.6, CH_2_	1.29, m	30.6, CH_2_	1.31, m
16	28.7, CH_2_	1.24, m	30.5, CH_2_	1.29, m	30.5, CH_2_	1.31, m
17	28.6, CH_2_	1.24, m	30.3, CH_2_	1.29, m	30.3, CH_2_	1.31, m
18	31.3, CH_2_	1.23, m	33.1, CH_2_	1.28, m	26.9, CH_2_	1.35, m
19	22.1, CH_2_	1.25, m	23.7, CH_2_	1.31, m	33.7, CH_2_	1.52, m
20	13.9, CH_3_	0.85, t (6.9)	14.4, CH_3_	0.90, t (7.0)	63.0, CH_2_	3.54, t (6.7)
3-OH		5.03, s				
4-OH		5.47, d (5.9)				
6-OH		4.74, t (5.0)				
7-OH		4.75, d (5.9)				

*^a^Recorded at 600 (^1^H) and 150 MHz (^13^C) in DMSO-d_6_. ^b^Recorded at 600 (^1^H) and 150 MHz (^13^C) in CD_3_OD.*

Aplosporellin B (**3**): Colorless oil; [α]^20^_*D*_ -55 (*c* 0.1, MeOH); UV (MeOH) λ_max_ 233 nm; ECD λ [nm] (ϕ): 233 (−7.06), 199 (−8.83); ^1^H and ^13^C NMR data, see Table 1; HRESIMS [M + Na]^+^ *m*/*z* 394.2197 (calcd for C_19_H_33_NNaO_6_ 394.2200) (Supplementary Figures S14–S21).

Aplosporellin C (**4**): Colorless oil; [α]^20^_D_ -26 (*c* 0.1, MeOH); UV (MeOH) λ_max_ 234 nm; ECD λ [nm] (ϕ): 233 (−0.98), 197 (−2.03); ^1^H and ^13^C NMR data, see Table 2; HRESIMS [M + Na]^+^ *m*/*z* 394.2201 (calcd for C_19_H_33_NNaO_6_ 394.2200) (Supplementary Figures S22–S29).

**TABLE 2:** ^1^H and ^13^C NMR data of compounds **4–6**.

No.	4*^*a*^*		5*^*a*^*		6*^*a*^*	
	**δ_*C*_, type**	**δ_*H*_ (*J* in Hz)**	**δ_*C*_, type**	**δ_*H*_ (*J* in Hz)**	**δ_*C*_, type**	**δ_*H*_ (*J* in Hz)**
2	177.3, C		177.4, C		177.4, C	
3	80.8, C		80.8, C		80.8, C	
4	77.9, CH	4.12, d (6.5)	78.0, CH	4.12, d (6.5)	78.0, CH	4.12, d (6.5)
5	61.2, CH	3.38, ddd (6.5, 5.8, 3.2)	61.2, CH	3.38, ddd (6.5, 5.8, 3.3)	61.2, CH	3.38, ddd (6.5, 5.8, 3.2)
6	62.9, CH_2_	3.76, dd (11.5, 3.2) 3.52, dd (11.5, 5.8)	63.0, CH_2_	3.76, dd (11.6, 3.3) 3.52, dd (11.6, 5.8)	63.0, CH_2_	3.76, dd (11.5, 3.2) 3.52, dd (11.5, 5.8)
7	74.5, CH	4.43, d (7.1)	74.6, CH	4.43, d (7.1)	74.5, CH	4.43, d (7.0)
8	129.6, CH	5.82, dd (15.3, 7.1)	129.6, CH	5.82, dd (15.3, 7.1)	129.7, CH	5.82, dd (15.3, 7.0)
9	134.6, CH	6.32, dd (15.3, 10.5)	134.6, CH	6.32, dd (15.3, 10.5)	134.6, CH	6.32, dd (15.3, 10.5)
10	131.2, CH	6.07, dd (15.0, 10.5)	131.2, CH	6.07, dd (15.0, 10.5)	131.2, CH	6.07, dd (15.0, 10.5)
11	136.3, CH	5.70, dt (15.0, 7.3)	136.3, CH	5.70, dt (15.0, 7.3)	136.3, CH	5.70, dt (15.0, 7.3)
12	33.7, CH_2_	2.08, q (7.3)	33.7, CH_2_	2.09, q (7.3)	33.7, CH_2_	2.08, q (7.3)
13	30.4, CH_2_	1.39, m	30.4, CH_2_	1.40, m	30.3, CH_2_	1.39, m
14	30.7, CH_2_	1.31, m	30.8, CH_2_	1.33, m	30.6, CH_2_	1.31, m
15	30.6, CH_2_	1.31, m	30.5, CH_2_	1.33, m	30.5, CH_2_	1.31, m
16	30.2, CH_2_	1.31, m	30.2, CH_2_	1.33, m	30.4, CH_2_	1.31, m
17	26.9, CH_2_	1.40, m 1.33, m	26.7, CH_2_	1.48, m 1.34, m	30.2, CH_2_	1.31, m
18	40.2, CH_2_	1.44, m 1.40, m	34.4, CH_2_	1.49, m 1.36, m	27.0, CH_2_	1.35, m
19	68.6, CH	3.70, m	73.3, CH	3.56, m	29.7, CH_2_	1.62, m
20	23.5, CH_3_	1.14, d (6.2)	67.4, CH_2_	3.46, dd (11.2, 4.5) 3.41, dd (11.2, 6.5)	65.7, CH_2_	4.05, t (6.7)
20-OAc					20.8, CH_3_ 173.1, C	2.02, s

*^a^Recorded at 600 (^1^H) and 150 MHz (^13^C) in CD_3_OD.*

Aplosporellin D (**5**): Colorless oil; [α]^20^_D_ -24 (*c* 0.1, MeOH); UV (MeOH) λ_max_ 234 nm; ^1^H and ^13^C NMR data, see Table 2; HRESIMS [M + Na]^+^ *m*/*z* 410.2145 (calcd for C_19_H_33_NNaO_7_ 410.2149) (Supplementary Figures S30–S37).

Aplosporellin E (**6**): Colorless oil; [α]^20^_D_ -33 (*c* 0.1, MeOH); UV (MeOH) λ_max_ 233 nm; ^1^H and ^13^C NMR data, see Table 2; HRESIMS [M + Na]^+^ *m*/*z* 436.2307 (calcd for C_21_H_35_NNaO_7_ 436.2306) (Supplementary Figures S38–S45).

Aplosporellin F (**7**): Colorless oil; [α]^20^_D_ -36 (*c* 0.1, MeOH); UV (MeOH) λ_max_ 234 nm; ^1^H and ^13^C NMR data, see Table 3; HRESIMS [M + Na]^+^ *m*/*z* 436.2304 (calcd for C_21_H_35_NNaO_7_ 436.2306) (Supplementary Figures S46–S53).

**TABLE 3:** ^1^H and ^13^C NMR data of compounds **7–9**.

No.	7*^*a*^*		8*^*a*^*		9*^*a*^*	
	**δ_*C*_, type**	**δ_*H*_ (*J* in Hz)**	**δ_*C*_, type**	**δ_*H*_ (*J* in Hz)**	**δ_*C*_, type**	**δ_*H*_ (*J* in Hz)**
2	177.3, C		177.3, C		177.3, C	
3	80.8, C		80.9, C		80.8, C	
4	77.9, CH	4.12, d (6.5)	77.9, CH	4.12, d (6.6)	78.0, CH	4.12, d (6.6)
5	61.2, CH	3.38, ddd (6.5, 5.8, 3.2)	61.1, CH	3.39, ddd (6.6, 5.8, 3.2)	61.2, CH	3.38, ddd (6.6, 5.8, 3.3)
6	62.9, CH_2_	3.76, dd (11.5, 3.2) 3.52, dd (11.5, 5.8)	62.9, CH_2_	3.76, dd (11.6, 3.2) 3.52, dd (11.6, 5.8)	63.0, CH_2_	3.76, dd (11.5, 3.3) 3.52, dd (11.5, 5.8)
7	74.5, CH	4.43, d (7.1)	74.5, CH	4.44, d (7.1)	74.6, CH	4.43, d (7.0)
8	129.6, CH	5.83, dd (15.3, 7.1)	129.5, CH	5.82, dd (15.3, 7.1)	129.7, CH	5.82, dd (15.4, 7.0)
9	134.6, CH	6.32, dd (15.3, 10.5)	134.6, CH	6.32, dd (15.3, 10.5)	134.6, CH	6.32, dd (15.4, 10.6)
10	131.2, CH	6.07, dd (15.1, 10.5)	131.2, CH	6.07, dd (15.0, 10.5)	131.2, CH	6.07, dd (15.0, 10.6)
11	136.3, CH	5.70, dt (15.1, 7.3)	136.3, CH	5.70, dt (15.0, 7.3)	136.3, CH	5.70, dt (15.0, 7.3)
12	33.6, CH_2_	2.08, q (7.3)	33.6, CH_2_	2.08, q (7.3)	33.7, CH_2_	2.08, q (7.3)
13	30.4, CH_2_	1.39, m	30.3, CH_2_	1.39, m	30.3, CH_2_	1.39, m
14	30.5, CH_2_	1.31, m	30.4, CH_2_	1.32, m	30.5, CH_2_	1.31, m
15	30.5, CH_2_	1.31, m	30.3, CH_2_	1.32, m	30.4, CH_2_	1.31, m
16	30.2, CH_2_	1.31, m	30.2, CH_2_	1.32, m	30.2, CH_2_	1.31, m
17	26.5, CH_2_	1.31, m	30.1, CH_2_	1.32, m	30.1, CH_2_	1.31, m
18	36.9, CH_2_	1.58, m 1.50, m	26.2, CH_2_	1.59, m	26.0, CH_2_	1.60, m
19	72.4, CH	4.86, m	35.2, CH_2_	2.27, t (7.4)	34.8, CH_2_	2.31, t (7.4)
20	20.2, CH_3_	1.20, d (6.2)	178.1, C		176.1, C	
19-OAc	21.2, CH_3_ 172.7, C	2.00, s				
20-OMe					52.0, CH_3_	3.65, s

*^a^Recorded at 600 (^1^H) and 150 MHz (^13^C) in CD_3_OD.*

Aplosporellin G (**8**): Colorless oil; [α]^20^_D_ -57 (*c* 0.1, MeOH); UV (MeOH) λ_max_ 233 nm; ECD λ [nm] (ϕ): 234 (−2.59), 197 (−7.64); ^1^H and ^13^C NMR data, see Table 3; HRESIMS [M + Na]^+^ *m*/*z* 408.1990 (calcd for C_19_H_31_NNaO_7_ 408.1993) (Supplementary Figures S54–S61).

Aplosporellin H (**9**): Colorless oil; [α]^20^_D_ -26 (*c* 0.1, MeOH); UV (MeOH) λ_max_ 232 nm; ^1^H and ^13^C NMR data, see Table 3; HRESIMS [M + Na]^+^ *m*/*z* 422.2150 (calcd for C_20_H_33_NNaO_7_ 422.2149) (Supplementary Figures S62–S69).

Aplosporellin I (**10**): Colorless oil; [α]^20^_D_ -31 (*c* 0.1, MeOH); UV (MeOH) λ_max_ 233 nm; ^1^H and ^13^C NMR data, see Table 4; HRESIMS [M + Na]^+^ *m*/*z* 450.2102 (calcd for C_21_H_33_NNaO_8_ 450.2098) (Supplementary Figures S70–S76).

**TABLE 4:** ^1^H and ^13^C NMR data of compounds **10–12.**

No.	10*^*a*^*		11*^*a*^*		12*^*a*^*	
	**δ_*C*_*^*b*^*, type**	**δ_*H*_ (*J* in Hz)**	**δ_*C*_, type**	**δ_*H*_ (*J* in Hz)**	**δ_*C*_*^*b*^*, type**	**δ_*H*_ (*J* in Hz)**
2	177.3, C		177.3, C		177.4, C	
3	80.5, C		80.8, C		80.6, C	
4	78.1, CH	4.10, d (6.8)	77.9, CH	4.12, d (6.5)	78.1, CH	4.10, d (6.7)
5	58.2, CH	3.55, ddd (6.8, 6.2, 3.3)	61.2, CH	3.38, ddd (6.5, 5.8, 3.2)	58.3, CH	3.55, ddd (6.7, 6.2, 3.4)
6	65.0, CH_2_	4.30, dd (11.6, 3.3) 4.05, dd (11.6, 6.2)	63.0, CH_2_	3.76, dd (11.6, 3.2) 3.52, dd (11.6, 5.8)	65.0, CH_2_	4.30, dd (11.7, 3.4) 4.04, dd (11.7, 6.2)
7	74.5, CH	4.42, d (7.0)	74.5, CH	4.43, d (7.1)	74.6, CH	4.42, d (7.1)
8	129.4, CH	5.82, dd (15.3, 7.0)	129.7, CH	5.82, dd (15.3, 7.1)	129.4, CH	5.82, dd (15.4, 7.1)
9	134.5, CH	6.32, dd (15.3, 10.5)	134.6, CH	6.32, dd (15.3, 10.5)	134.5, CH	6.32, dd (15.4, 10.6)
10	131.2, CH	6.07, dd (15.1, 10.5)	131.2, CH	6.07, dd (15.0, 10.5)	131.2, CH	6.07, dd (15.0, 10.6)
11	136.3, CH	5.70, dt (15.1, 7.3)	136.3, CH	5.70, dt (15.0, 7.3)	136.3, CH	5.71, dt (15.0, 7.3)
12	33.6, CH_2_	2.08, q (7.3)	33.6, CH_2_	2.08, q (7.3)	33.6, CH_2_	2.08, q (7.3)
13	30.2, CH_2_	1.39, m	30.2, CH_2_	1.39, m	30.3, CH_2_	1.40, m
14	30.4, CH_2_	1.32, m	30.3, CH_2_	1.31, m	30.6, CH_2_	1.31, m
15	30.3, CH_2_	1.32, m	30.2, CH_2_	1.31, m	30.5, CH_2_	1.31, m
16	30.2, CH_2_	1.32, m	30.1, CH_2_	1.31, m	30.4, CH_2_	1.31, m
17	30.1, CH_2_	1.32, m	24.8, CH_2_	1.54, m	30.2, CH_2_	1.31, m
18	26.7, CH_2_	1.59, m	44.3, CH_2_	2.47, t (7.3)	27.0, CH_2_	1.35, m
19	36.6, CH_2_	2.22, t (7.4)	212.3, C		29.6, CH_2_	1.62, m
20	179.8, C		29.8, CH_3_	2.13, s	65.7, CH_2_	4.05, t (6.7)
6-OAc	20.5, CH_3_ 172.5, C	2.07, s			20.6, CH_3_ 172.4, C	2.07, s
20-OAc					20.8, CH_3_ 173.0, C	2.02, s

*^a^Recorded at 600 (^1^H) and 150 MHz (^13^C) in CD_3_OD.^b^Data extracted from HSQC and HMBC spectra.*

Aplosporellin J (**11**): Colorless oil; [α]^20^_D_ -7 (*c* 0.1, MeOH); UV (MeOH) λ_max_ 234 nm; ^1^H and ^13^C NMR data, see Table 4; HRESIMS [M + Na]^+^ *m*/*z* 392.2048 (calcd for C_19_H_31_NNaO_6_ 392.2044) (Supplementary Figures S77–S84).

Aplosporellin K (**12**): Colorless oil; [α]^20^_D_ -17 (*c* 0.1, MeOH); UV (MeOH) λ_max_ 232 nm; ^1^H and ^13^C NMR data, see Table 4; HRESIMS [M + Na]^+^ *m*/*z* 478.2410 (calcd for C_23_H_37_NNaO_8_ 478.2411) (Supplementary Figures S85–S91).

### Cytotoxicity and Apoptosis Assays

Cytotoxicity against adult Burkitt’s lymphoma B cells (Ramos, No. ACC-603) and lymphoblastic leukemia T cells (Jurkat J16, No. ACC-282) was tested as described previously (Harwoko et al., 2019). In the apoptosis assays, the protein kinase inhibitor staurosporine (STS, 2.5 μM, #S5921, Sigma-Aldrich) was used as positive control, and standard growth medium supplemented with 0.1% DMSO was used as negative control. Determination of cell viability, western blot analysis and measurement of caspase-3 activity were performed as described previously (Manns et al., 2011; Liu et al., 2017). All experiments were carried out in triplicate.

### Computational Methods

Mixed torsional/low-mode conformational searches were carried out by means of the Macromodel 10.8.011 software using the MMFF with an implicit solvent model for CHCl_3_ applying a 21 kJ mol^–1^ energy window (MacroModel, 2015). Geometry reoptimizations of the resultant conformers [B3LYP/6-31 + G(d,p) level *in vacuo*, ωB97X/TZVP with PCM solvent model for MeCN and MeOH], DFT-NMR, TDDFT-ECD and SOR calculations were performed with Gaussian 09 (Frisch et al., 2013). For NMR calculations the mPW1PW91/6-311 + G(2d,p) level while for the ECD and SOR calculations the B3LYP/TZVP, BH&HLYP/TZVP, CAM-B3LYP/TZVP and PBE0/TZVP levels were applied with the same or no solvent model as in the preceding DFT optimization level. ECD spectra were generated as the sum of Gaussians with 4200 and 3000 cm^–1^ half-height widths, using dipole-velocity-computed rotational strengths (Stephens and Harada, 2010). Computed NMR shift data were corrected with I = −185.6277 and S = −1.0175 (Pierens, 2014). Boltzmann distributions were estimated from the B3LYP and the ωB97X energies. The MOLEKEL program was used for visualization of the results (Varetto, 2009).

## Results

### Chemical Identification of the Isolated Compounds

**FIGURE 3:**
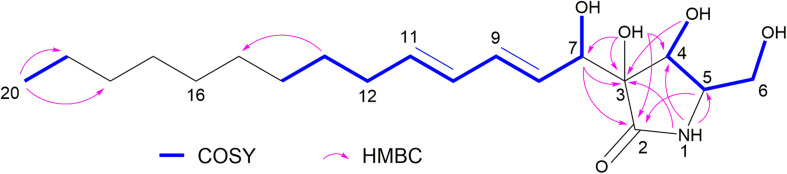
COSY and key HMBC correlations of compound 2.

Compound **2** was obtained as a colorless oil, with UV absorption at λ_max_ 233 nm. Its molecular formula was established as C_19_H_33_NO_5_ on the basis of HRESIMS data, accounting for four degrees of unsaturation. The NMR spectra of **2** (Table 1) were recorded in methanol-*d*_4_ as well as in DMSO-*d*_6_. The latter solvent revealed the exchangeable protons of one NH proton at δ_H_ 7.69 (NH-1) as well as four OH protons at δ_H_ 5.47 (4-OH), 5.03 (3-OH), 4.75 (7-OH), and 4.74 (6-OH). The ^13^C NMR spectrum of **2** displayed one carbonyl group at δ_C_ 174.4 (C-2), and four olefinic carbons at δ_C_ 133.6 (C-11), 131.0 (C-9), 130.8 (C-8), and 130.2 (C-10), accounting for three degrees of unsaturation. The presence of a γ-lactam ring was confirmed by the COSY correlations between 4-OH/H-4 (δ_H_ 3.89)/H-5 (δ_H_ 3.16)/H_2_-6 (δ_H_ 3.54 and 3.30)/6-OH and the HMBC correlations from NH-1 to C-3, C-4, and C-5, from 3-OH to C-2, C-3 and C-4, from 4-OH to C-3, and from H-5 to C-2 (Figure 3). The COSY correlations between H-8 (δ_H_ 5.82)/H-9 (δ_H_ 6.14)/H-10 (δ_H_ 6.00)/H-11 (δ_H_ 5.62)/H_2_-12 (δ_H_ 2.04)/H_2_-13 (δ_H_ 1.34)/H_2_-14 (δ_H_ 1.24), and between Me-20 (δ_H_ 0.85)/H_2_-19 (δ_H_ 1.24) together with the HMBC correlations from Me-20 to C-19 (δ_C_ 22.1) and C-18 (δ_C_ 31.3) and the observation of characteristic aliphatic methylenes at 29.0, 28.9, 28.7, 28.6, and δ_H_ 1.24 (CH_2_-14, 15, 16, and 17), established the presence of a trideca-1,3-diene subunit in **2**. In addition, the COSY correlations between H-8/H-7 (δ_H_ 4.22)/7-OH along with the HMBC correlations from H-7 to C-2, C-3, and C-4, and from 3-OH to C-7 indicated the trideca-1,3-diene side chain to be connected to the γ-lactam moiety via the oxygenated carbon at C-7. Therefore, the planar structure of **2** was elucidated, which was similar to that of the co-isolated known compound, pramanicin A (**1**) (Schwartz et al., 1994; Harrison et al., 2000). The major difference between both compounds was the presence of an additional hydroxy group in **2** instead of the ketone group in **1** at position C-7. The trivial name aplosporellin A is proposed for **2**.

The large values of *J*_8_,_9_ (15.4 Hz) and *J*_10_,_11_ (15.1 Hz) supported the *E* configuration for the double bonds at C-8/C-9 and C-10/C-11 in **2**. The relative configuration of the γ-lactam ring was deduced through the ROESY spectrum and by comparison with pramanicin A (**1**) (Harrison et al., 2000), virgaricins A and B (Ishii et al., 2012, 2015). The NOE correlations between 3-OH/H-4, H-4/H_2_-6 indicated that 3-OH, H-4 and H_2_-6 were located on the same side of the ring. In contrast, the NOE correlations between H-7/4-OH, 4-OH/H-5, indicated that 4-OH and H-5 were located on the opposite side compared to 3-OH, H-4 and H_2_-6. On the basis of these findings, the relative stereochemistry of **2** in the γ-lactam moiety was assigned to be identical to that of pramanicin A (**1**).

**FIGURE 4:**
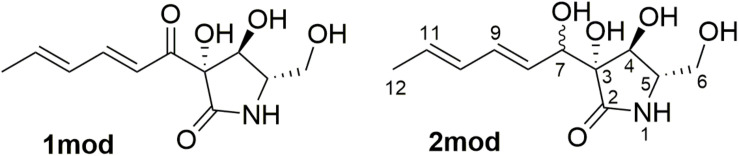
Truncated model compounds applied for the SOR, NMR and ECD calculations.

Pramanicin A (**1**) was reported together with pramanicin from the fungus *Stagonospora* sp. (Schwartz et al., 1994), the latter contained an epoxide group at C-10/C-11 instead of a double bond. The absolute configuration of pramanicin A (**1**) was deduced by comparison to pramanicin through biogenetic considerations (Duspara et al., 1998; Harrison et al., 1998, 2000; Chen and Harrison, 2004) and by total synthesis (Cow et al., 1997; Barrett et al., 1999a, b; Tan et al., 2014, 2015, 2017), which confirmed that the compound has (3*S*,4*S*,5*S*) configuration. The large absolute value of the specific optical rotation (SOR) of **1** allowed testing the TDDFT-SOR method (Polavarapu, 2002; Mándi and Kurtán, 2019) and the ωB97X functional (Chai and Head-Gordon, 2008; Bremond et al., 2016), which was also applied for the TDDFT-ECD calculations of **2**. Merck Molecular Force Field (MMFF) conformational searches of **1** and the epimers of **2** resulted in a large number of conformers [c.a. 18 thousand by generating 100 thousand structures for (3*R*,4*S*,5*S*,7*S*)-**2**] and the searches were not complete, since many conformers were found only a few times (Mándi et al., 2015). Thus, model compounds were utilized for the calculations, in which the C-3 side-chain was truncated at the C-12 position (Figure 4).

The MMFF conformational search of (3*S*,4*S*,5*S*)-**1mod** resulted in 79 conformers in a 21 kJ/mol energy window, which were re-optimized at the ωB97X/TZVP PCM/MeOH level yielding 16 low-energy conformers over 1% Boltzmann-distribution (Supplementary Figure S92). SOR values were computed at four levels (B3LYP/TZVP, BH&HLYP/TZVP, CAM-B3LYP/TZVP and PBE0/TZVP all with the PCM solvent model for MeOH) and nice agreements were found with the experimental SOR value (computed SOR values in the range from −91 to −115 compared to the −121 experimental value) (Supplementary Table S1).

**FIGURE 5:**
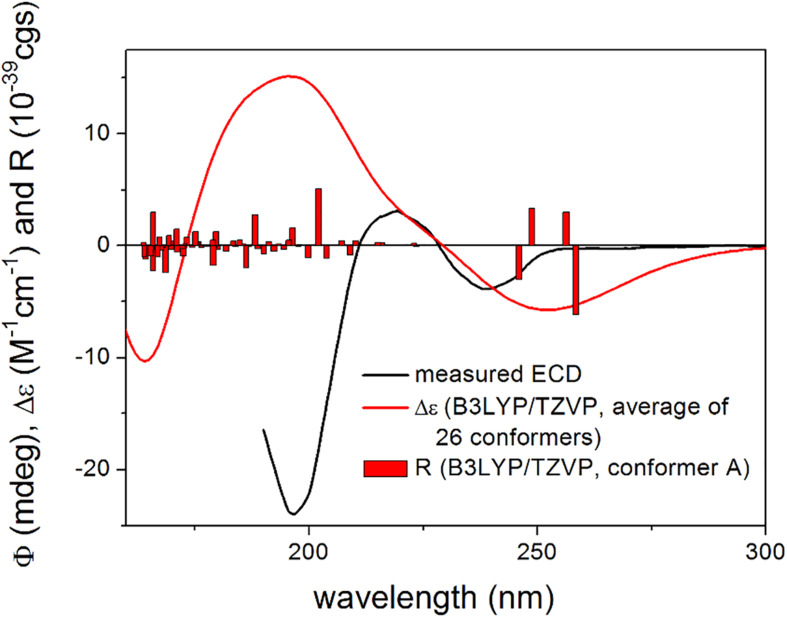
Experimental ECD spectrum of 2 in MeCN compared with the Boltzmann-weighted B3LYP/TZVP PCM/MeCN ECD spectrum of (3*R*,4*S*,5*S*,7*S*)-**2mod**. Level of optimization: ωB97X/TZVP PCM/MeCN. Bars represent the rotatory strength values of the lowest-energy conformer. The experimental spectrum was scaled to the computed one.

The absolute configuration of the γ-lactam ring of compound **2** is assumed to be identical to that of **1** based on biogenetic considerations whereas that of the OH group in the side chain at C-7 could not be elucidated. In order to solve this problem, DFT-NMR calculations were performed on the epimeric model compounds (3*R*,4*S*,5*S*,7*R*)-**2mod** and (3*R*,4*S*,5*S*,7*S*)-**2mod** (Lodewyk et al., 2012; Kicsák et al., 2018; Mándi and Kurtán, 2019). B3LYP/6-31 + G(d,p) re-optimization of the initial 169 and 205 conformers resulted in 8 and 15 low-energy conformers over 1% Boltzmann population, respectively (Supplementary Figures S93, S94). The ^13^C NMR chemical shift data of (3*R*,4*S*,5*S*,7*S*)-**2mod** computed at the mPW1PW91/6-311 + G(2d,p) level reproduced much better the experimental values than those of the other epimer (Supplementary Table S2). Mean absolute error (MAE) values were 1.70 vs. 2.17 favoring the (3*R*,4*S*,5*S*,7*S*) epimer. The signal of the terminal C-12 was neglected, since it has an abnormal shift in the computations due to the truncation. Computed and the experimental ^13^C NMR chemical shift data in the vicinity of the C-7 chirality center also clearly showed superior agreement for the (3*R*,4*S*,5*S*,7*S*) epimer. DP4 + statistical analysis was utilized for the comparison of the experimental and calculated ^13^C NMR data resulting in 97.31% confidence for the (3*R*,4*S*,5*S*,7*S*) epimer (Smith and Goodman, 2010; Grimblat et al., 2015; Li et al., 2020). Although the experimental SOR value of **2** is small, the SOR calculations performed the same way as for **1mod** were in agreement with the results of the NMR calculation suggesting also (3*R*,4*S*,5*S*,7*S*) absolute configuration. The (3*R*,4*S*,5*S*,7*R*) epimer of **2mod** gave computed SOR values ranging from +4 to +7 while the (3*R*,4*S*,5*S*,7*S*) one in the range from −18 to −23 reproducing better the −21 experimental value of **2** (Supplementary Tables S3, S4). For the TDDFT-ECD method, the same MMFF conformers of (3*R*,4*S*,5*S*,7*R*)-**2mod** and (3*R*,4*S*,5*S*,7*S*)-**2mod** were re-optimized at the ωB97X/TZVP PCM/MeCN level and ECD calculations were performed at various levels. Although both epimers gave rather diverse computed ECD spectra for the individual conformers and Boltzmann populations were small, the average ECD spectra of the (3*R*,4*S*,5*S*,7*S*) epimer were found to be similar to the experimental one (Figure 5) in line with the NMR and the SOR calculations. Consequently, the absolute configuration of **2** could be elucidated as (3*R*,4*S*,5*S*,7*S*).

The molecular formula of compound **3** was assigned as C_19_H_33_NO_6_ based on its HRESIMS data, containing an additional oxygen atom when compared to **2**. The NMR data of **3** (Table 1) were similar to those of **2**, except for the presence of an oxygenated methylene group resonating at δ_C_ 63.0 and δ_H_ 3.54 (t) (CH_2_-20) and the absence of the terminal methyl group in the side chain. The COSY correlation between H_2_-20/H_2_-19 (δ_H_ 1.52)/H_2_-18 (δ_H_ 1.35) together with the HMBC correlations from H_2_-20 to C-18 (δ_C_ 26.9) and C-19 (δ_*C*_ 33.7) indicated the attachment of a hydroxy group at C-20 in the side chain of **3**. Detailed analysis of its 2D NMR spectra revealed that the remaining substructure of **3** was identical to that of **2**. The absolute configuration of **3** was identical to that of **2** based on their similar ROESY correlations and ECD data.

Aplosporellin C (**4**) exhibited the same molecular formula as **3** as determined by HRESIMS data. The ^1^H and ^13^C NMR data of **4** (Table 2) were likewise similar to those of **3**, yet showed the signal of a doublet methyl group at δ_C_ 23.5 and δ_H_ 1.14 (Me-20) in the side chain and the presence of an oxygenated methine at δ_C_ 68.6 and δ_H_ 3.70 (CH-19). The COSY correlations between Me-20/H-19, and between H-19/H_2_-18 (δ_*H*_ 1.44 and 1.40) together with the HMBC correlations from Me-20 to C-19 and C-18 (δ_*C*_ 40.2) indicated the presence of a hydroxy group at C-19 in **4** instead of C-20 as in **3**. The remaining substructure of **4** was identical to that of **2** as confirmed by detailed interpretation of the 2D NMR spectra of **4**. Due to the limited amount, the absolute configuration at C-19 of **4** could not be determined by converting the compound to its Mosher ester.

Compound **5** had the molecular formula C_19_H_33_NO_7_ as determined by HRESIMS data, containing one additional oxygen atom when compared to **3** and **4**. Detailed analysis of the 2D NMR of **5** (Table 2) revealed that it was similar to **3** except for the presence of one additional oxygenated methine at δ_C_ 73.3 and δ_H_ 3.56 (CH-19). The signal of the terminal oxygenated methylene at C-20 appeared as two dd peaks in the ^1^H NMR spectrum of **5** instead of a triplet peak of **3**. This suggested that the additional hydroxy group of **5** was located at C-19, which was further confirmed by the COSY correlations between H_2_-20/H-19/H_2_-18 together with the HMBC correlations from H_2_-20 to C-19 and C-18. Thus, compound **5** was elucidated as 19,20-dihydroxy derivative of **2**.

Aplosporellin E (**6**) was found to have the molecular formula C_21_H_35_NO_7_ on the basis of HRESIMS data, requiring five degrees of unsaturation. Comparison of the NMR data (Table 2) indicated compound **6** to be closely related to compound **3** except for the presence of an additional methyl group (δ_H_ 2.02 and δ_C_ 20.8) and an additional carbonyl carbon (δ_C_ 173.1). The HMBC correlations from protons of the additional methyl and H_2_-20 (δ_H_ 4.05) to the additional carbonyl carbon indicated the attachment of an additional acetoxy group at C-20 in **6** when compared to **3**.

The molecular formula of compound **7** was the same as **6** as deduced from HRESIMS. The ^1^H and ^13^C NMR data of **7** (Table 3) were very similar to those of **6**, except for the appearance of a terminal methyl group at δ_H_ 1.20 (Me-20) which was split into a doublet peak in the ^1^H NMR spectrum of **7**. Detailed analysis of the HSQC and HMBC spectra revealed an oxygenated methine at δ_C_ 72.4 and δ_H_ 4.86 (CH-19). The COSY correlations between Me-20/H-19/H_2_-18 (δ_*H*_ 1.58 and 1.50) together with the HMBC correlations from H-19 and the methyl group at δ_H_ 2.00 (3H, s) to the carbonyl carbon at δ_C_ 172.7 indicated the presence of an acetoxy group at C-19. Thus, compound **7** was elucidated as 19-*O*-acetyl derivative of compound **4**.

Compound **8** exhibited the molecular formula C_19_H_31_NO_7_ as determined by the HRESIMS data. Its ^1^H and ^13^C NMR data (Table 3) were similar to those of **2** except for that signals of the terminal methyl group in the side chain was replaced with a carbonyl group at δ_C_ 178.1 (C-20). The HMBC correlations from H_2_-18 (δ_H_ 1.59) and H_2_-19 (δ_H_ 2.27, t) to C-20, together with the COSY correlations between H_2_-18/H_2_-19 indicated a terminal carboxylic acid group in the side chain. The remaining substructure of **8** was identical to that of **2** as confirmed by detailed analysis of the 2D NMR spectra of **8**.

The HRESIMS data of **9** gave the molecular formula C_20_H_33_NO_7_. From the 2D NMR spectra of **9** (Table 3) it was evident that compound **9** was the 20-*O*-methyl derivative of **8** as indicated by the presence of an additional methoxy group at δ_H_ 3.65 (3H, s) and δ_C_ 52.0, together with the HMBC correlations from the methoxy group and H_2_-19 (δ_H_ 2.31, t) to the carbonyl carbon at δ_C_ 176.1 (C-20).

Aplosporellin I (**10**) exhibited the molecular formula C_21_H_33_NO_8_ as determined by HRESIMS, requiring six degrees of unsaturation. The NMR data of **10** (Table 4) were similar to those of **8** except for the presence of a methyl group (δ_H_ 2.07 and δ_C_ 20.5) and a carbonyl carbon (δ_C_ 172.5) in addition to minor differences of the chemical shifts of the protons at the γ-lactam ring moiety. The COSY correlations between H-4 (δ_H_ 4.10)/H-5 (δ_H_ 3.55)/H_2_-6 (δ_H_ 4.30 and 4.05) together with the HMBC correlations from H_2_-6 and the additional methyl group at δ_H_ 2.07 to the carbonyl carbon at δ_C_ 172.5 indicated the attachment of an acetoxy group at C-6. Detailed analysis of the 2D NMR spectra and the ROESY spectra of compound **10** revealed that the remaining substructure and relative configuration were identical to compound **8.** Thus, compound **10** was identified as the 6-*O*-acetyl derivative of **8**.

The molecular formula of **11** was determined as C_19_H_31_NO_6_ based on HRESIMS data, accounting for five degrees of unsaturation. The NMR data of **11** (Table 4) were similar to those of **2** but exhibited the signal of an additional carbonyl group at δ_C_ 212.3 (C-19). Moreover, the methyl group in the side chain was shifted to down field at δ_C_ 29.8, δ_H_ 2.13 (Me-20) and appeared as singlet in the ^1^H NMR spectrum. The HMBC correlations from Me-20, H_2_-18 (δ_H_ 2.47) and H_2_-17 (δ_H_ 1.54) to C-19 indicated the presence of a ketone group in the side chain at C-19 in **11**. The remaining substructure of **11** was identical to that of **2** as confirmed by detailed analysis of the 2D NMR spectra.

On the basis of the HRESIMS data, the molecular formula of **12** was established as C_23_H_37_NO_8_ with six degrees of unsaturation. The NMR data of **12** (Table 4) were similar to those of **6**, except for the presence of an additional methyl group (δ_H_ 2.07 and δ_C_ 20.6) and a carbonyl carbon (δ_C_ 172.4). Moreover, the chemical shifts of the protons of the γ-lactam ring in **12** were more comparable to those of **10** rather than **6**. These findings suggested compound **12** was a 6-*O*-acetyl derivative of **6**, which was confirmed by the HMBC correlations from H_2_-6 (δ_H_ 4.30 and δ_C_ 4.04) and the additional methyl group at δ_H_ 2.07 to the carbonyl carbon at δ_C_ 172.4.

### Bioactivities of the Isolated Compounds

**FIGURE 6:**
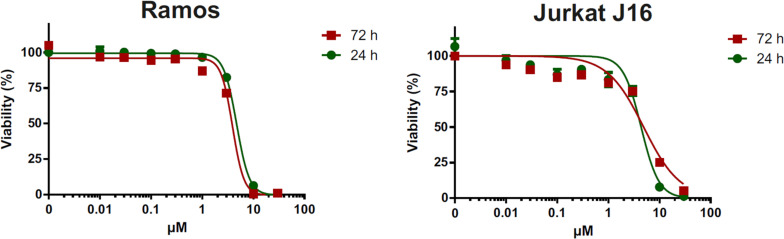
Alamar blue assay of pramanicin A (**1**) in Ramos and Jurkat J16 cells. Each cell line was treated with 0–300 μM pramanicin A (**1**) for 24 or 72 h and after incubation, cell viability was evaluated using Alamar blue assay. Results shown are the mean ± SD from triplicates.

All isolated compounds were tested for their cytotoxicity against human lymphoma (Ramos) and leukemia (Jurkat J16) cell lines. Pramanicin A (**1**) exhibited IC_50_ values of 4.7 and 4.4 μM after 24 h of incubation respectively, whereas after 72 h of incubation these values were 3.9 and 4.9 μM, respectively. Treatment with pramanicin A (**1**) significantly affected cell viability in a dose-dependent manner (Figure 6) whereas the remaining compounds showed no cytotoxicity in these two cell lines in the observed concentration ranging up to 30 μM. The ketone group at C-7 of pramanicin A (**1**) which is conjugated to the olefinic function at C-8/C-9 is obviously the key factor responsible for the cytotoxicity against human Ramos and Jurkat J16 cell lines. It was suggested that the α,β-unsaturated ketone functionality in pramanicin A (**1**) could be Michael acceptors that can react with a thiol group of cysteine amino acid of proteins or enzymes, and thus causing cytotoxic activity (Amslinger, 2010; Darsih et al., 2015).

**FIGURE 7:**
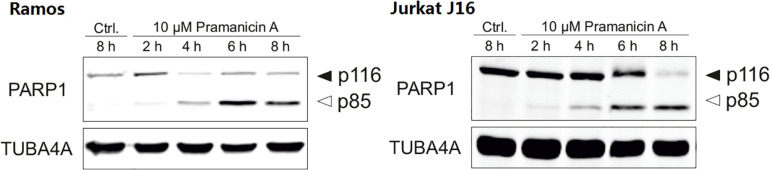
Western blot experiment of pramanicin A (**1**) in Ramos and Jurkat J16 cells. After incubation for 2–8 h, cleavage of PARP1 was determined. Solid arrowheads indicate the uncleaved form of PARP1, open arrowheads indicate the cleaved form. The expression of TUBA4A was determined as protein loading control. Negative control (Ctrl.) was 0.1% DMSO.

**FIGURE 8:**
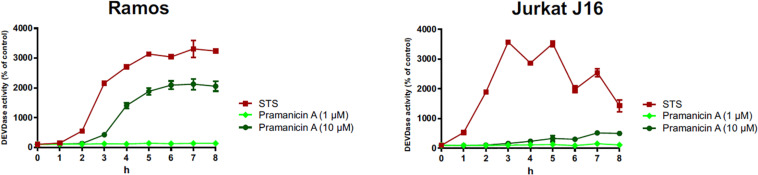
Caspase-3 assay: the kinetics of caspase-3 activation in Ramos and Jurkat J16 cells after treatment with indicated concentrations of pramanicin A (**1**). Caspase-3 activity was measured by the rate of cleavage of Ac-DEVD-AMC. Cells treated with staurosporine (STS, 2.5 μM) were used as positive control. Cells treated with 0.1% DMSO were used as negative control and set to 100%. Results shown are the mean ± SD from triplicates.

In order to evaluate whether the pronounced cytotoxicity of pramanicin A (**1**) is attributable to the induction of apoptosis, we followed the activation of the effector caspases such as caspase-3 in response to pramanicin A (**1**) treatment. In the Western blot experiment, after 2–8 h treatment of Ramos and Jurkat J16 cell lines with 10 μM pramanicin A (**1**) respectively, an explicit cleavage of PARP1 (poly [ADP-ribose] polymerase 1) was observed (Figure 7). Cleavage of PARP1, which is a substrate of caspase-3, serves as a surrogate marker for activation of caspase-3 and therefore indicated that pramanicin A (**1**) is able to induce apoptosis. Moreover, we also measured caspase-3 activity by detecting the profluorogenic caspase-3 substrate Ac-DEVD-AMC. After treatment with 10 μM pramanicin A (**1**) in the two cell lines, cleavage of Ac-DEVD-AMC was observed within a few hours, which was more obvious in Ramos cells than in Jurkat J16 cells (Figure 8). These results further proved the activation of caspase-3 and thus induction of apoptosis in Ramos and Jurkat J16 cells by pramanicin A (**1**).

## Discussion

In the previous biosynthetic study on pramanicin, which differs from pramanicin A (**1**) by the presence of an epoxide group at C-10/C-11 instead of the double bond, in the fungus *Stagonospora* sp., Duspara et al. (1998) and Harrison et al. (2000) conducted a feeding experiment using ^2^H, ^13^C, ^15^N, and ^18^O isotopically labeled precursors. It was concluded that pramanicin originates from one starter molecule acetate and six extender malonates to generate the aliphatic acyl tail, whereas L-serine interacts with one acetate to form the pyrrolidone ring. Acetylation of these two moieties provides 3-acyltetramic acid, followed by a series of oxidation and reduction reactions to form pramanicin (Harrison et al., 2000). Proline or glutamate on the other hand were shown not be precursors of the pyrrolidone moiety (Harrison et al., 1998). In our study, the addition of 3.5% NaNO_3_ or of 3.5% C_5_H_8_NNaO_4_⋅H_2_O to solid rice medium was found to induce the accumulation of pramanicin-like compounds. Thus, a feeding study using labeled glutamate as a potential precursor of compounds **1**–**12** would be of interest as a follow up study of this investigation.

## Conclusion

In summary, 11 new lactam derivatives, aplosporellins A–K (**2**–**12**), together with the known analog, pramanicin A (**1**), were isolated from fermentation of *A. javeedii* on solid rice medium with addition of either 3.5% NaNO_3_ or 3.5% monosodium glutamate. All of these compounds were not detected when the fungus was grown on rice medium without these activators. To the best of our knowledge, this study was the first report to regulate secondary metabolites from *A. javeedii* applying an OSMAC approach. The results proved the power of the OSMAC approach on mining new secondary metabolites from endophytic fungi. DFT-NMR, TDDFT-ECD and OR calculations were carried out to determine the absolute configuration. Pramanicin A (**1**) exhibited strong cytotoxicity against human lymphoma (Ramos) and leukemia (Jurkat J16) cell lines with IC_50_ values of 4.7 and 4.4 μM, respectively. Furthermore, mechanistic studies indicated that pramanicin A (**1**) activates caspase-3 and induces apoptotic cell death.

## Data Availability Statement

The original contributions presented in the study are included in the article/Supplementary Material, further inquiries can be directed to the corresponding authors.

## Author Contributions

YG sentence contributed to extraction, isolation, and manuscript preparation. FS, LS, and SW carried out the cytotoxicity assay. ZG and KZ contributed to part of structure elucidation. AM and TK performed the DFT-NMR, TDDFT-ECD, and OR calculations. ZL and PP supervised the research work and revised the manuscript. All the authors contributed to the article and approved the submitted version.

## Conflict of Interest

The authors declare that the research was conducted in the absence of any commercial or financial relationships that could be construed as a potential conflict of interest.
