# Supplementary material for: Induction of New Lactam Derivatives From the Endophytic Fungus Aplosporella javeedii Through an OSMAC Approach
Source: Front Microbiol. 2020 Nov 4;11:600983. doi: 10.3389/fmicb.2020.600983 (PMC7672018; doi:10.3389/fmicb.2020.600983)
Supplement: Supplementary file 1 [file Data_Sheet_1.DOCX]

Supplementary Material

**Induction of New Lactam Derivatives from the Endophytic Fungus *Aplosporella* *javeedii* through an OSMAC Approach**

*Ying Gao,^1^ Fabian Stuhldreier,^2^ Laura Schmitt,^2^ Sebastian Wesselborg,^2^ Zhiyong Guo,^3^ Kun Zou,^3^ Attila Mándi,^4^ Tibor Kurtán,^4^ Zhen Liu,^1,^* and Peter Proksch ^1,3,^**

*^1^Institute of Pharmaceutical Biology and Biotechnology, Heinrich Heine University Düsseldorf, Düsseldorf, Germany.*

*^2^Institute of Molecular Medicine I, Medical Faculty, Heinrich Heine University Düsseldorf, Düsseldorf, Germany.*

*^3^Hubei Key Laboratory of Natural Products Research and Development, College of Biological and Pharmaceutical Sciences, China Three Gorges University, Yichang, China.*

*^4^Department of Organic Chemistry, University of Debrecen, Debrecen, Hungary.*

**^*^Correspondence:**

Zhen Liu

zhenfeizi0@sina.com

Peter Proksch

proksch@uni-duesseldorf.de

**Table of Contents**

[**Figure S1.** HPLC chromatogram of compound **2** 6](#_Toc49110692)

[**Figure S2.** ^1^H NMR (600M Hz, DMSO-*d*_6_) spectrum of compound **2** 6](#_Toc49110693)

[**Figure S3.** ^13^C NMR (150M Hz, DMSO-*d*_6_) spectrum of compound **2** 7](#_Toc49110694)

[**Figure S4.** HSQC (DMSO-*d*_6_) spectrum of compound **2** 7](#_Toc49110695)

[**Figure S5.** COSY (DMSO-*d*_6_) spectrum of compound **2** 8](#_Toc49110696)

[**Figure S6.** HMBC (DMSO-*d*_6_) spectrum of compound **2** 8](#_Toc49110697)

[**Figure S7.** ROESY (DMSO-*d*_6_) spectrum of compound **2** 9](#_Toc49110698)

[**Figure S8.** ^1^H NMR (600M Hz, methanol-*d*_4_) spectrum of compound **2** 9](#_Toc49110699)

[**Figure S9.** ^13^C NMR (150M Hz, methanol-*d*_4_) spectrum of compound **2** 10](#_Toc49110700)

[**Figure S10.** HSQC (methanol-*d*_4_) spectrum of compound **2** 10](#_Toc49110701)

[**Figure S11.** COSY (methanol-*d*_4_) spectrum of compound **2** 11](#_Toc49110702)

[**Figure S12.** HMBC (methanol-*d*_4_) spectrum of compound **2** 11](#_Toc49110703)

[**Figure S13.** HRESIMS of compound **2** 12](#_Toc49110704)

[**Figure S14.** HPLC chromatogram of compound **3** 12](#_Toc49110705)

[**Figure S15.** ^1^H NMR (600M Hz, methanol-*d*_4_) spectrum of compound **3** 13](#_Toc49110706)

[**Figure S16.** ^13^C NMR (150M Hz, methanol-*d*_4_) spectrum of compound **3** 13](#_Toc49110707)

[**Figure S17.** HSQC (methanol-*d*_4_) spectrum of compound **3** 14](#_Toc49110708)

[**Figure S18.** COSY (methanol-*d*_4_) spectrum of compound **3** 14](#_Toc49110709)

[**Figure S19.** HMBC (methanol-*d*_4_) spectrum of compound **3** 15](#_Toc49110710)

[**Figure S20.** ROESY (methanol-*d*_4_) spectrum of compound **3** 15](#_Toc49110711)

[**Figure S21.** HRESIMS of compound **3** 16](#_Toc49110712)

[**Figure S22.** HPLC chromatogram of compound **4** 16](#_Toc49110713)

[**Figure S23.** ^1^H NMR (600M Hz, methanol-*d*_4_) spectrum of compound **4** 17](#_Toc49110714)

[**Figure S24.** ^13^C NMR (150M Hz, methanol-*d*_4_) spectrum of compound **4** 17](#_Toc49110715)

[**Figure S25.** HSQC (methanol-*d*_4_) spectrum of compound **4** 18](#_Toc49110716)

[**Figure S26.** COSY (methanol-*d*_4_) spectrum of compound **4** 18](#_Toc49110717)

[**Figure S27.** HMBC (methanol-*d*_4_) spectrum of compound **4** 19](#_Toc49110718)

[**Figure S28.** ROESY (methanol-*d*_4_) spectrum of compound **4** 19](#_Toc49110719)

[**Figure S29.** HRESIMS of compound **4** 20](#_Toc49110720)

[**Figure S30.** HPLC chromatogram of compound **5** 20](#_Toc49110721)

[**Figure S31.** ^1^H NMR (600M Hz, methanol-*d*_4_) spectrum of compound **5** 21](#_Toc49110722)

[**Figure S32.** ^13^C NMR (150M Hz, methanol-*d*_4_) spectrum of compound **5** 21](#_Toc49110723)

[**Figure S33.** HSQC (methanol-*d*_4_) spectrum of compound **5** 22](#_Toc49110724)

[**Figure S34.** COSY (methanol-*d*_4_) spectrum of compound **5** 22](#_Toc49110725)

[**Figure S35.** HMBC (methanol-*d*_4_) spectrum of compound **5** 23](#_Toc49110726)

[**Figure S36.** ROESY (methanol-*d*_4_) spectrum of compound **5** 23](#_Toc49110727)

[**Figure S37.** HRESIMS of compound **5** 24](#_Toc49110728)

[**Figure S38.** HPLC chromatogram of compound **6** 24](#_Toc49110729)

[**Figure S39.** ^1^H NMR (600M Hz, methanol-*d*_4_) spectrum of compound **6** 25](#_Toc49110730)

[**Figure S40.** ^13^C NMR (150M Hz, methanol-*d*_4_) spectrum of compound **6** 25](#_Toc49110731)

[**Figure S41.** HSQC (methanol-*d*_4_) spectrum of compound **6** 26](#_Toc49110732)

[**Figure S42.** COSY (methanol-*d*_4_) spectrum of compound **6** 26](#_Toc49110733)

[**Figure S43.** HMBC (methanol-*d*_4_) spectrum of compound **6** 27](#_Toc49110734)

[**Figure S44.** ROESY (methanol-*d*_4_) spectrum of compound **6** 27](#_Toc49110735)

[**Figure S45.** HRESIMS of compound **6** 28](#_Toc49110736)

[**Figure S46.** HPLC chromatogram of compound **7** 28](#_Toc49110737)

[**Figure S47.** ^1^H NMR (600M Hz, methanol-*d*_4_) spectrum of compound **7** 29](#_Toc49110738)

[**Figure S48.** ^13^C NMR (150M Hz, methanol-*d*_4_) spectrum of compound **7** 29](#_Toc49110739)

[**Figure S49.** HSQC (methanol-*d*_4_) spectrum of compound **7** 30](#_Toc49110740)

[**Figure S50.** COSY (methanol-*d*_4_) spectrum of compound **7** 30](#_Toc49110741)

[**Figure S51.** HMBC (methanol-*d*_4_) spectrum of compound **7** 31](#_Toc49110742)

[**Figure S52.** ROESY (methanol-*d*_4_) spectrum of compound **7** 31](#_Toc49110743)

[**Figure S53.** HRESIMS of compound **7** 32](#_Toc49110744)

[**Figure S54.** HPLC chromatogram of compound **8** 32](#_Toc49110745)

[**Figure S55.** ^1^H NMR (600M Hz, methanol-*d*_4_) spectrum of compound **8** 33](#_Toc49110746)

[**Figure S56.** ^13^C NMR (150M Hz, methanol-*d*_4_) spectrum of compound **8** 33](#_Toc49110747)

[**Figure S57.** HSQC (methanol-*d*_4_) spectrum of compound **8** 34](#_Toc49110748)

[**Figure S58.** COSY (methanol-*d*_4_) spectrum of compound **8** 34](#_Toc49110749)

[**Figure S59.** HMBC (methanol-*d*_4_) spectrum of compound **8** 35](#_Toc49110750)

[**Figure S60.** ROESY (methanol-*d*_4_) spectrum of compound **8** 35](#_Toc49110751)

[**Figure S61.** HRESIMS of compound **8** 36](#_Toc49110752)

[**Figure S62.** HPLC chromatogram of compound **9** 36](#_Toc49110753)

[**Figure S63.** ^1^H NMR (600M Hz, methanol-*d*_4_) spectrum of compound **9** 37](#_Toc49110754)

[**Figure S64.** ^13^C NMR (150M Hz, methanol-*d*_4_) spectrum of compound **9** 37](#_Toc49110755)

[**Figure S65.** HSQC (methanol-*d*_4_) spectrum of compound **9** 38](#_Toc49110756)

[**Figure S66.** COSY (methanol-*d*_4_) spectrum of compound **9** 38](#_Toc49110757)

[**Figure S67.** HMBC (methanol-*d*_4_) spectrum of compound **9** 39](#_Toc49110758)

[**Figure S68.** ROESY (methanol-*d*_4_) spectrum of compound **9** 39](#_Toc49110759)

[**Figure S69.** HRESIMS of compound **9** 40](#_Toc49110760)

[**Figure S70.** HPLC chromatogram of compound **10** 40](#_Toc49110761)

[**Figure S71.** ^1^H NMR (600M Hz, methanol-*d*_4_) spectrum of compound **10** 41](#_Toc49110762)

[**Figure S72.** HSQC (methanol-*d*_4_) spectrum of compound **10** 41](#_Toc49110763)

[**Figure S73.** COSY (methanol-*d*_4_) spectrum of compound **10** 42](#_Toc49110764)

[**Figure S74.** HMBC (methanol-*d*_4_) spectrum of compound **10** 42](#_Toc49110765)

[**Figure S75.** ROESY (methanol-*d*_4_) spectrum of compound **10** 43](#_Toc49110766)

[**Figure S76.** HRESIMS of compound **10** 43](#_Toc49110767)

[**Figure S77.** HPLC chromatogram of compound **11** 44](#_Toc49110768)

[**Figure S78.** ^1^H NMR (600M Hz, methanol-*d*_4_) spectrum of compound **11** 44](#_Toc49110769)

[**Figure S79.** ^13^C NMR (150M Hz, methanol-*d*_4_) spectrum of compound **11** 45](#_Toc49110770)

[**Figure S80.** HSQC (methanol-*d*_4_) spectrum of compound **11** 45](#_Toc49110771)

[**Figure S81.** COSY (methanol-*d*_4_) spectrum of compound **11** 46](#_Toc49110772)

[**Figure S82.** HMBC (methanol-*d*_4_) spectrum of compound **11** 46](#_Toc49110773)

[**Figure S83.** ROESY (methanol-*d*_4_) spectrum of compound **11** 47](#_Toc49110774)

[**Figure S84.** HRESIMS of compound **11** 47](#_Toc49110775)

[**Figure S85.** HPLC chromatogram of compound **12** 48](#_Toc49110776)

[**Figure S86.** ^1^H NMR (600M Hz, methanol-*d*_4_) spectrum of compound **12** 48](#_Toc49110777)

[**Figure S87.** HSQC (methanol-*d*_4_) spectrum of compound **12** 49](#_Toc49110778)

[**Figure S88.** COSY (methanol-*d*_4_) spectrum of compound **12** 49](#_Toc49110779)

[**Figure S89.** HMBC (methanol-*d*_4_) spectrum of compound **12** 50](#_Toc49110780)

[**Figure S90.** ROESY (methanol-*d*_4_) spectrum of compound **12** 50](#_Toc49110781)

[**Figure S91.** HRESIMS of compound **12** 51](#_Toc49110782)

[**Figure S92.** Low-energy conformers and populations of (3*S*,4*S*,5*S*)-**1mod** computed at the ωB97X/TZVP PCM/MeOH level of theory. 52](#_Toc49110783)

[**Figure S93.** Low-energy conformers and populations of (3*R*,4*S*,5*S*,7*R*)-**2mod** computed at the B3LYP/6-31+G(d,p) level of theory. 53](#_Toc49110784)

[**Figure S94.** Low-energy conformers and populations of (3*R*,4*S*,5*S*,7*S*)-**2mod** computed at the B3LYP/6-31+G(d,p) level of theory. 53](#_Toc49110785)

[**Figure S95.** Experimental ECD spectrum of **2** in MeCN compared with the Boltzmann-weighted B3LYP/TZVP PCM/MeCN ECD spectrum of (3*R*,4*S*,5*S*,7*R*)-**2mod**. 54](#_Toc49110786)

[**Figure S96.** Low-energy conformers and populations of (3*R*,4*S*,5*S*,7*R*)-**2mod** computed at the ωB97X/TZVP PCM/MeCN level of theory. 55](#_Toc49110787)

[**Figure S97.** Low-energy conformers and populations of (3*R*,4*S*,5*S*,7*S*)-**2mod** computed at the ωB97X/TZVP PCM/MeCN level of theory. 56](#_Toc49110788)

[**Table S1.** Computed SOR values for (3*S*,4*S*,5*S*)-**1mod** at various levels. 57](#_Toc49110789)

[**Table S2.** Comparison of the experimental ^13^C NMR data of the carbons of the **2** measured in MeOH-*d*_4_ with the mPW1PW91/6-311+G(2d,p) // B3LYP/6-31+G(d,p) ones of the (3*R*,4*S*,5*S*,7*R*)-**2mod** and (3*R*,4*S*,5*S*,7*S*)-**2mod** stereoisomers. 58](#_Toc49110790)

[**Table S3.** Computed SOR values for (3*R*,4*S*,5*S*,7*R*)-**2mod** at various levels. 59](#_Toc49110791)

[**Table S4.** Computed SOR values for (3*R*,4*S*,5*S*,7*S*)-**2mod** at various levels. 60](#_Toc49110792)

**Figure S1.** HPLC chromatogram of compound **2**

UV absorption of compound **2**

**Figure S2.** ^1^H NMR (600M Hz, DMSO-*d*_6_) spectrum of compound **2**


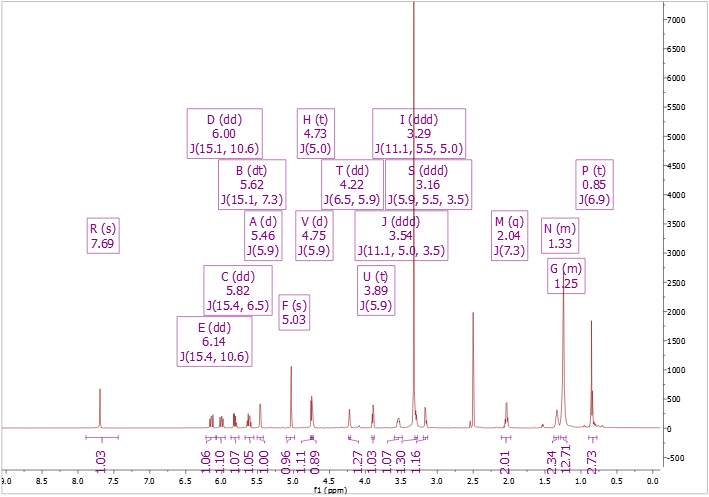


**Figure S3.** ^13^C NMR (150M Hz, DMSO-*d*_6_) spectrum of compound **2**


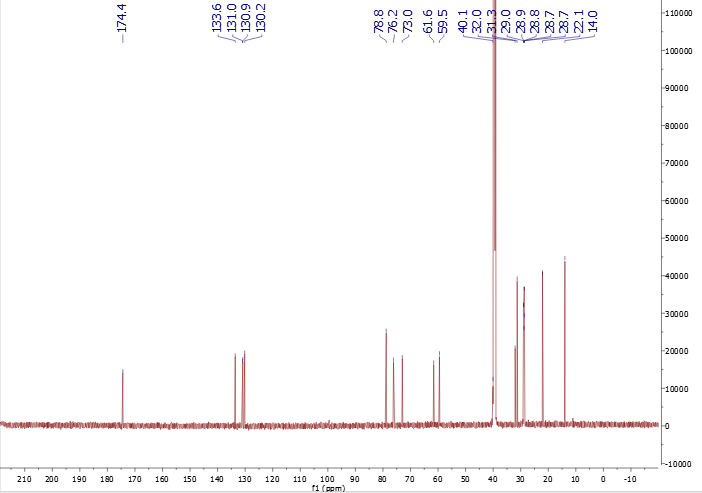


**Figure S4.** HSQC (DMSO-*d*_6_) spectrum of compound **2**


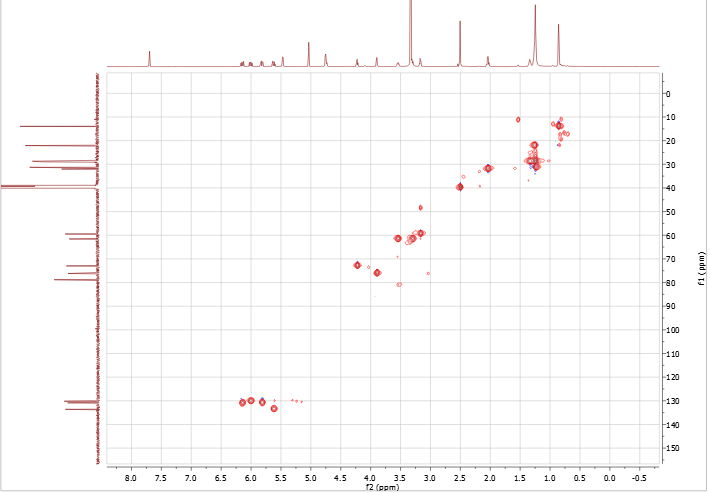


**Figure S5.** COSY (DMSO-*d*_6_) spectrum of compound **2**


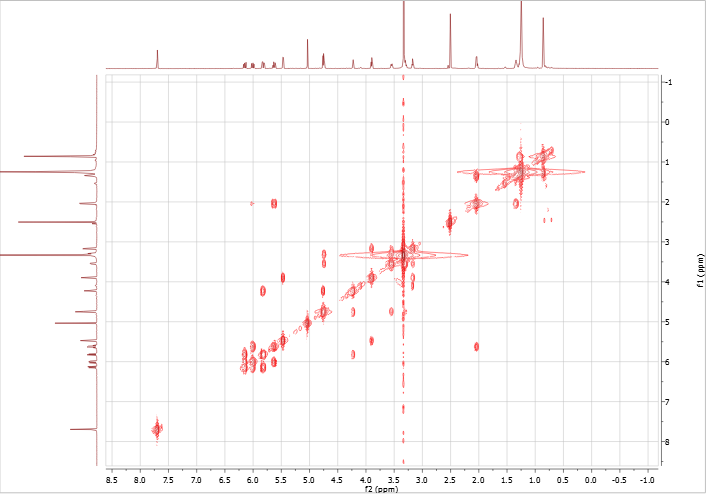


**Figure S6.** HMBC (DMSO-*d*_6_) spectrum of compound **2**


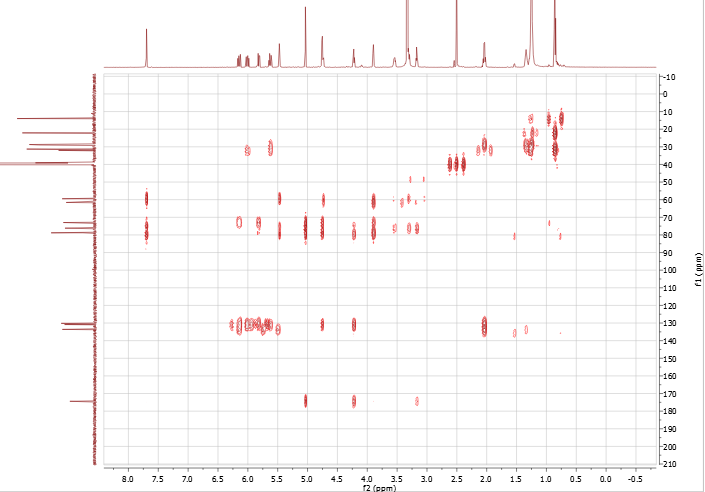


**Figure S7.** ROESY (DMSO-*d*_6_) spectrum of compound **2**


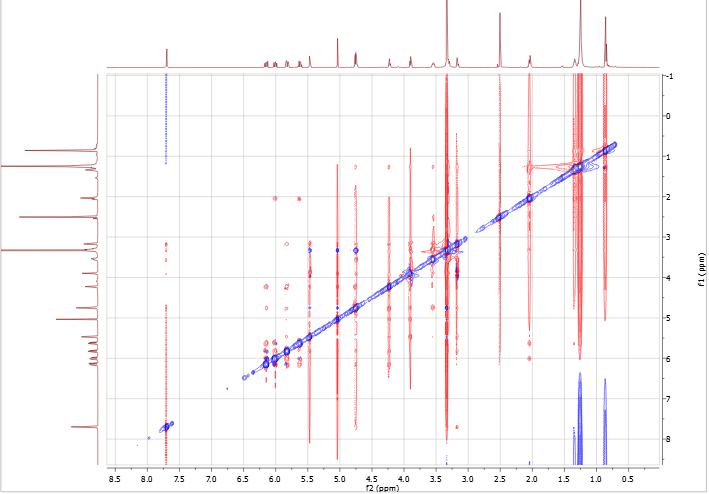


**Figure S8.** ^1^H NMR (600M Hz, methanol-*d*_4_) spectrum of compound **2**


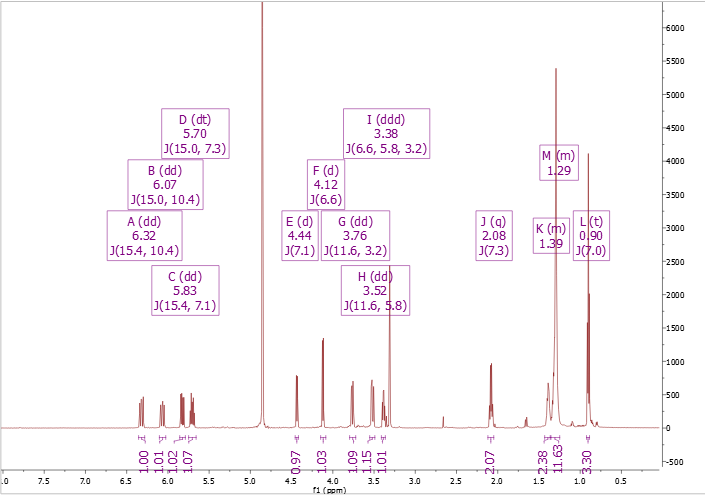


**Figure S9.** ^13^C NMR (150M Hz, methanol-*d*_4_) spectrum of compound **2**


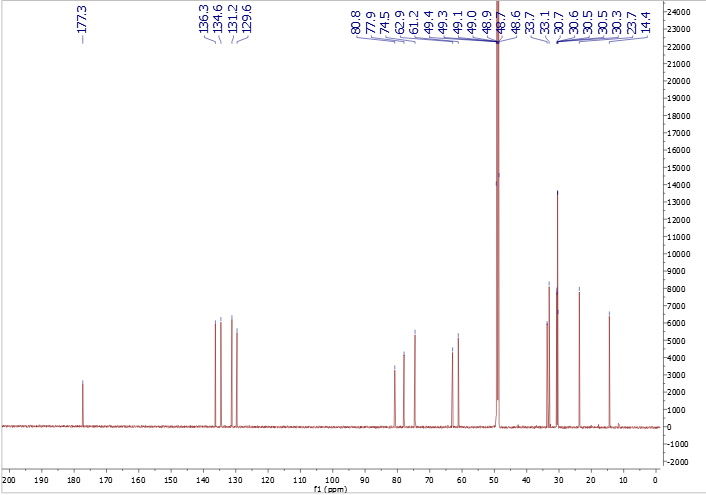


**Figure S10.** HSQC (methanol-*d*_4_) spectrum of compound **2**


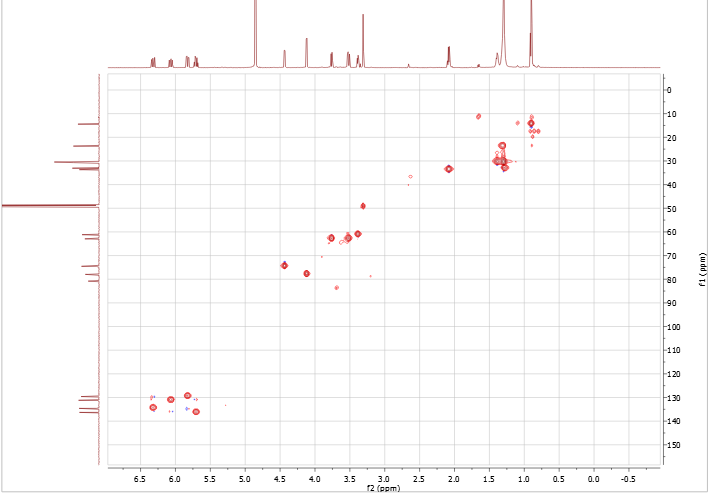


**Figure S11.** COSY (methanol-*d*_4_) spectrum of compound **2**


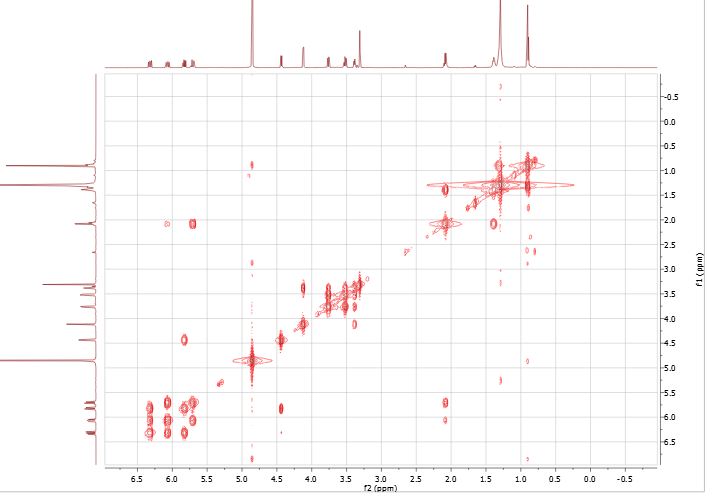


**Figure S12.** HMBC (methanol-*d*_4_) spectrum of compound **2**


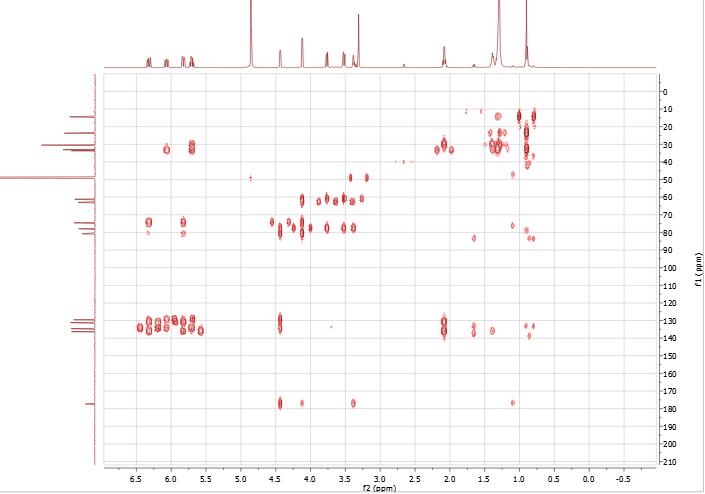


**Figure S13.** HRESIMS of compound **2**


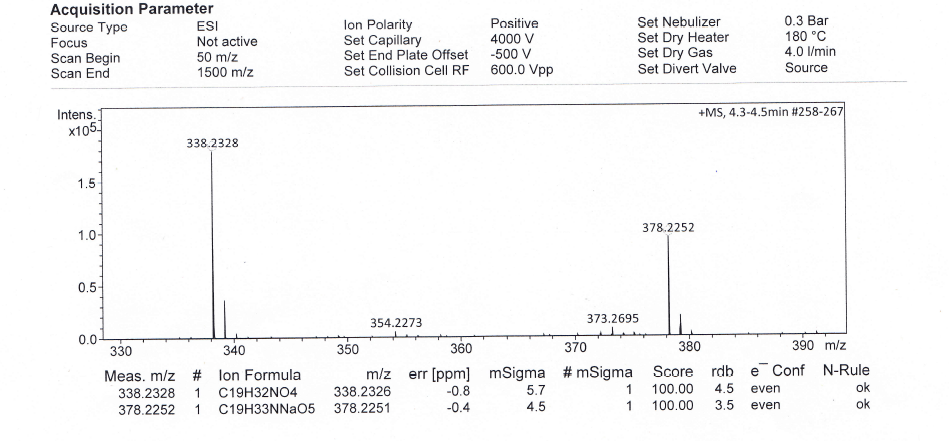


**Figure S14.** HPLC chromatogram of compound **3**

UV absorption of compound **3**

**Figure S15.** ^1^H NMR (600M Hz, methanol-*d*_4_) spectrum of compound **3**


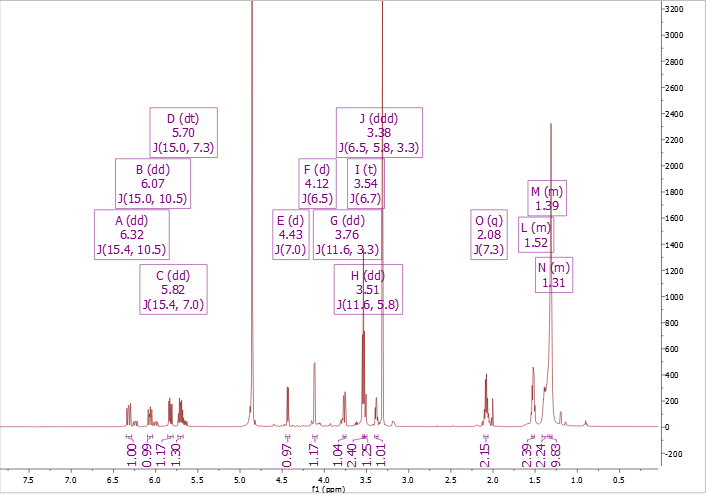


**Figure S16.** ^13^C NMR (150M Hz, methanol-*d*_4_) spectrum of compound **3**


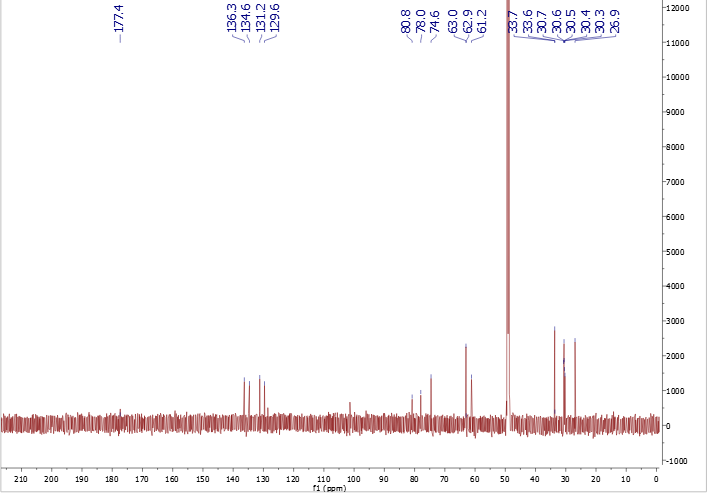


**Figure S17.** HSQC (methanol-*d*_4_) spectrum of compound **3**


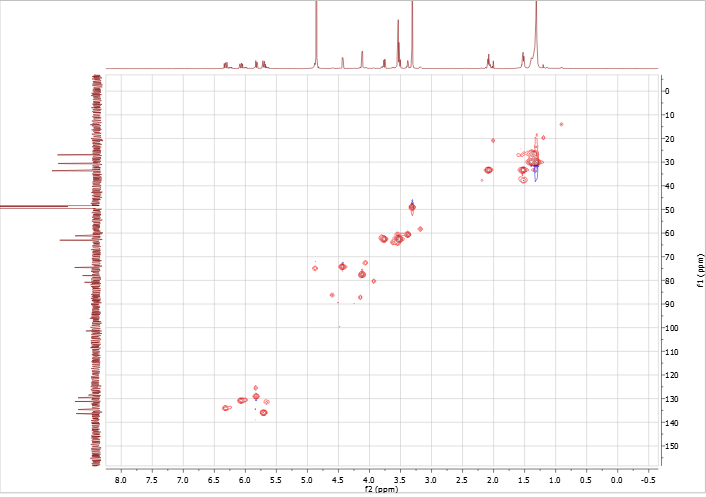


**Figure S18.** COSY (methanol-*d*_4_) spectrum of compound **3**


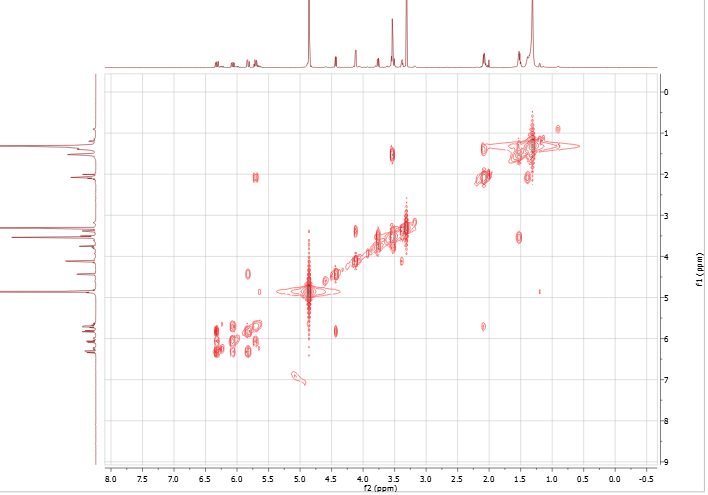


**Figure S19.** HMBC (methanol-*d*_4_) spectrum of compound **3**


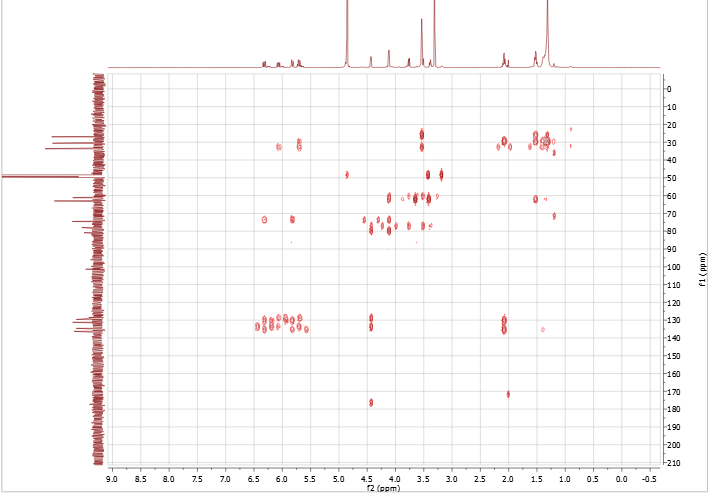


**Figure S20.** ROESY (methanol-*d*_4_) spectrum of compound **3**


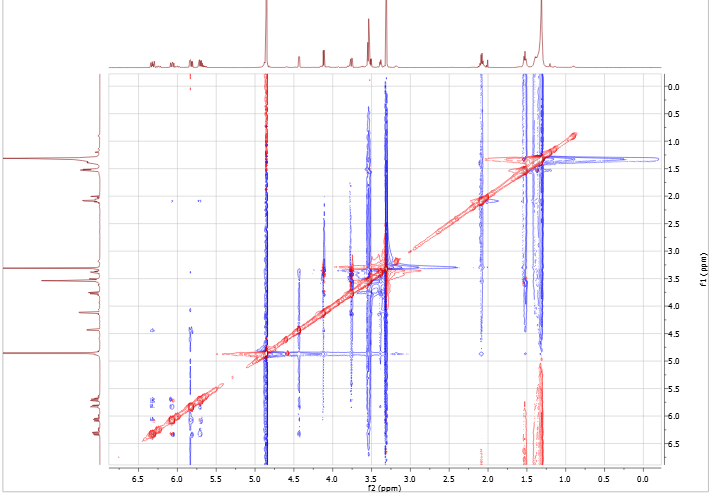


**Figure S21.** HRESIMS of compound **3**


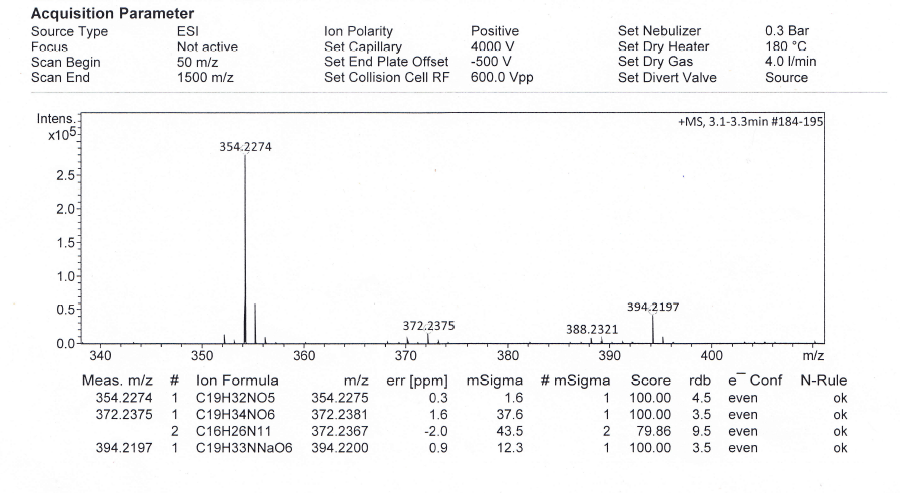


**Figure S22.** HPLC chromatogram of compound **4**

UV absorption of compound **4**

**Figure S23.** ^1^H NMR (600M Hz, methanol-*d*_4_) spectrum of compound **4**


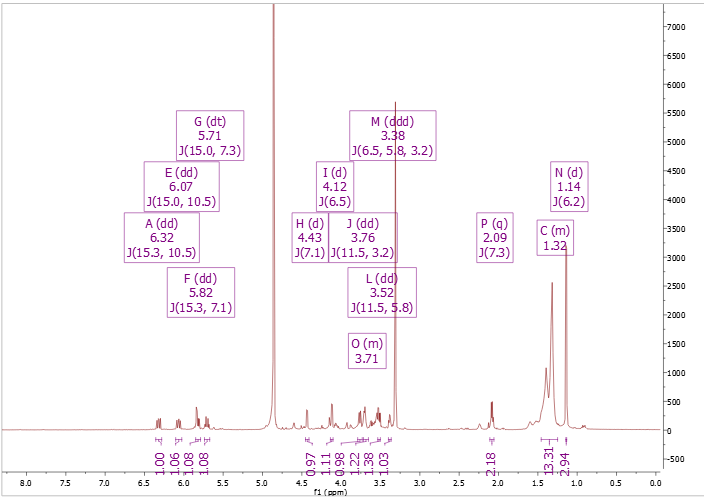


**Figure S24.** ^13^C NMR (150M Hz, methanol-*d*_4_) spectrum of compound **4**


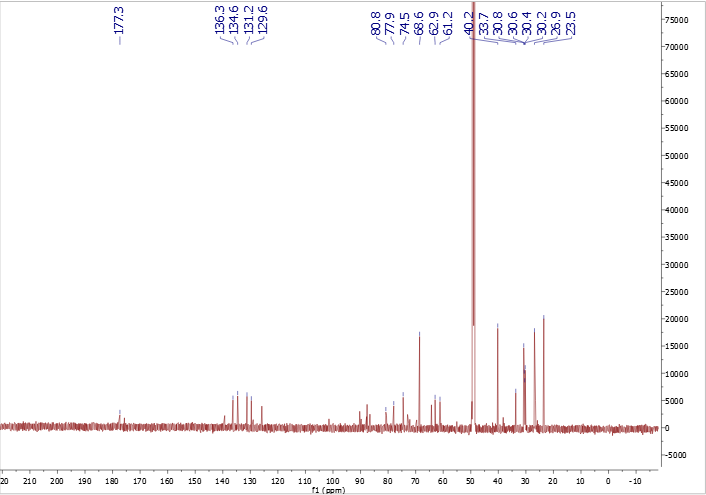


**Figure S25.** HSQC (methanol-*d*_4_) spectrum of compound **4**


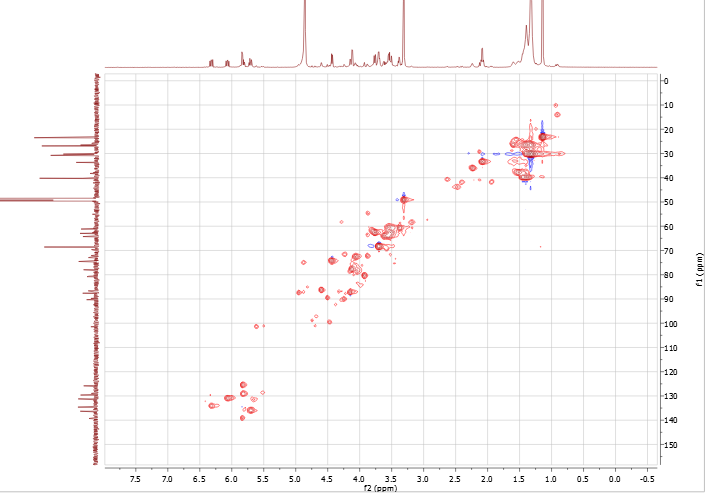


**Figure S26.** COSY (methanol-*d*_4_) spectrum of compound **4**


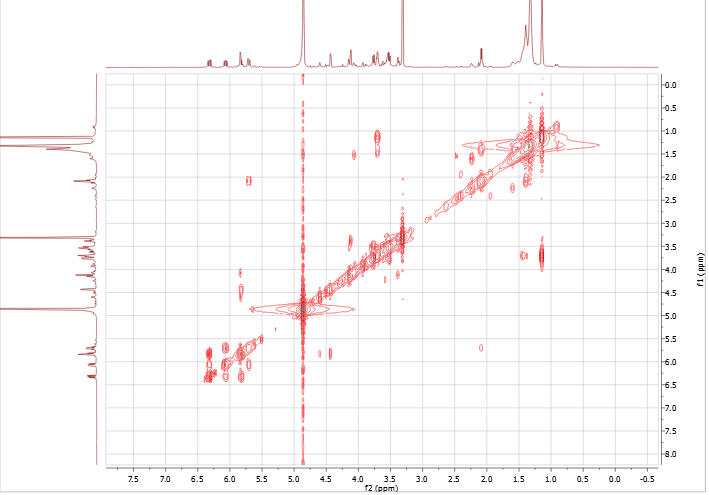


**Figure S27.** HMBC (methanol-*d*_4_) spectrum of compound **4**


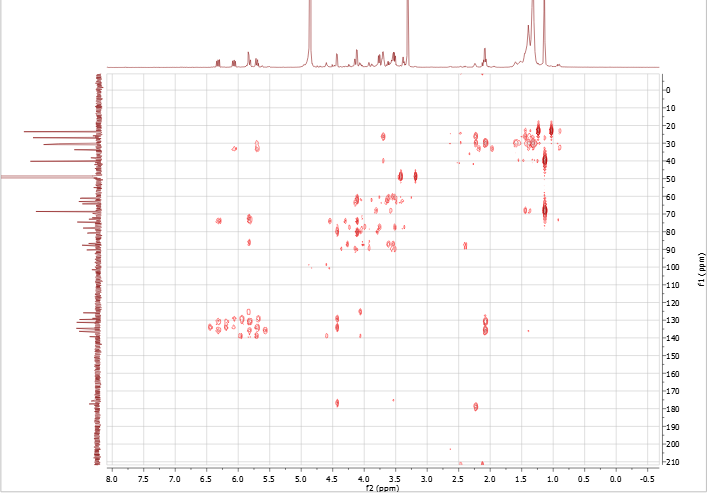


**Figure S28.** ROESY (methanol-*d*_4_) spectrum of compound **4**


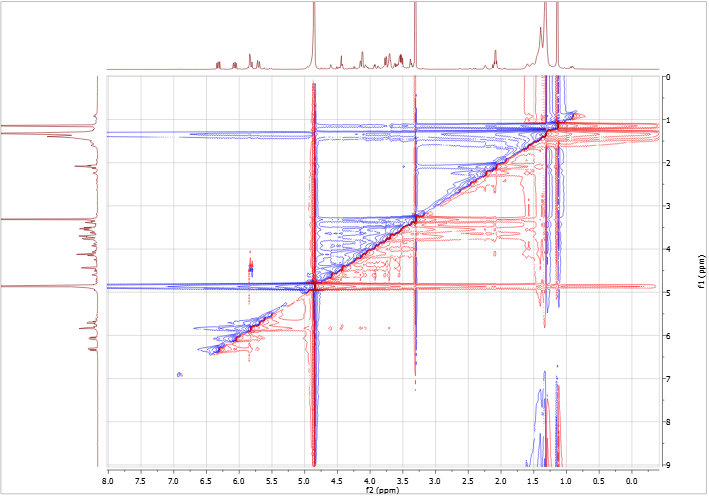


**Figure S29.** HRESIMS of compound **4**


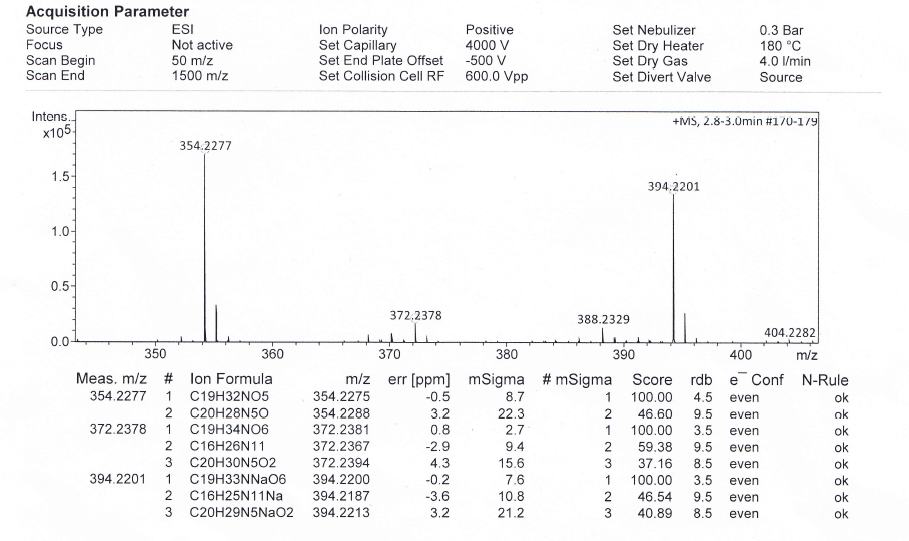


**Figure S30.** HPLC chromatogram of compound **5**

UV absorption of compound **5**

**Figure S31.** ^1^H NMR (600M Hz, methanol-*d*_4_) spectrum of compound **5**


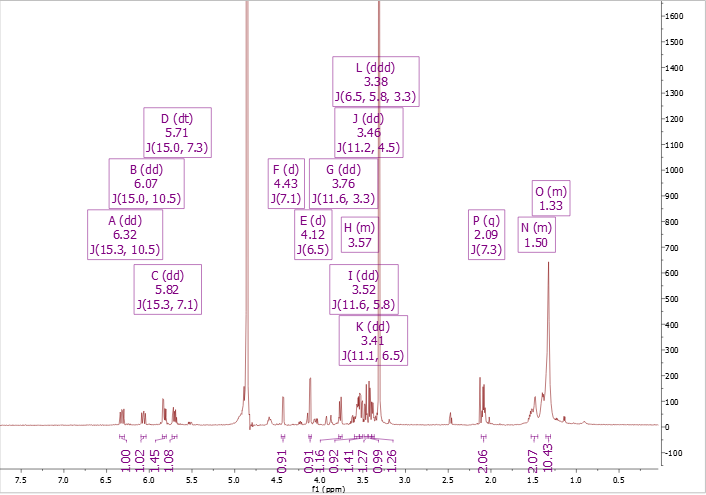


**Figure S32.** ^13^C NMR (150M Hz, methanol-*d*_4_) spectrum of compound **5**


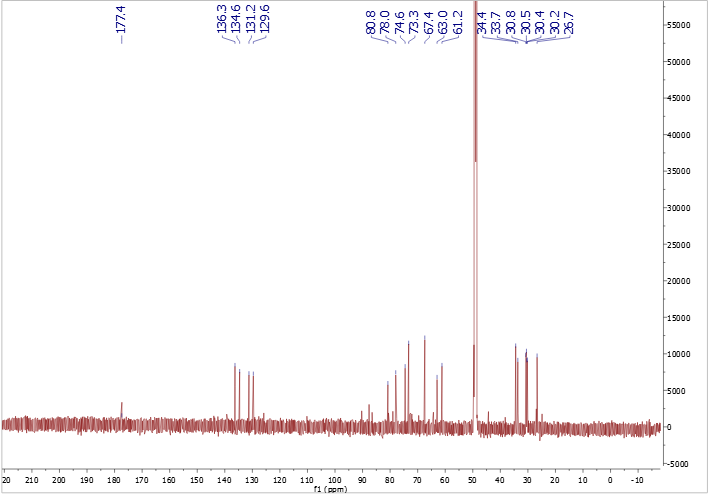


**Figure S33.** HSQC (methanol-*d*_4_) spectrum of compound **5**


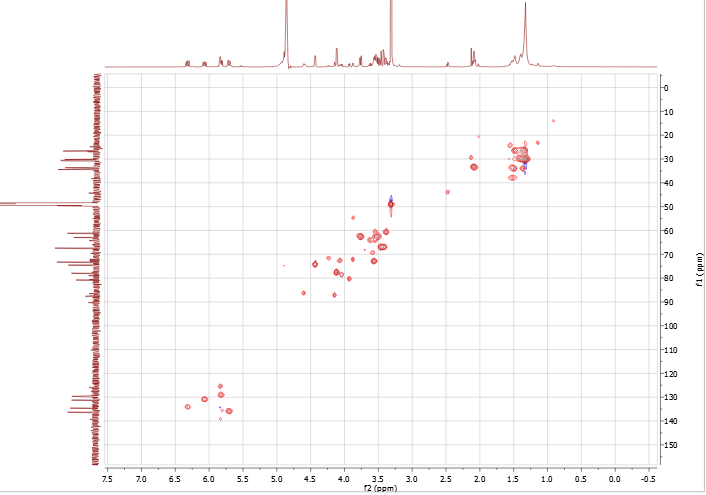


**Figure S34.** COSY (methanol-*d*_4_) spectrum of compound **5**


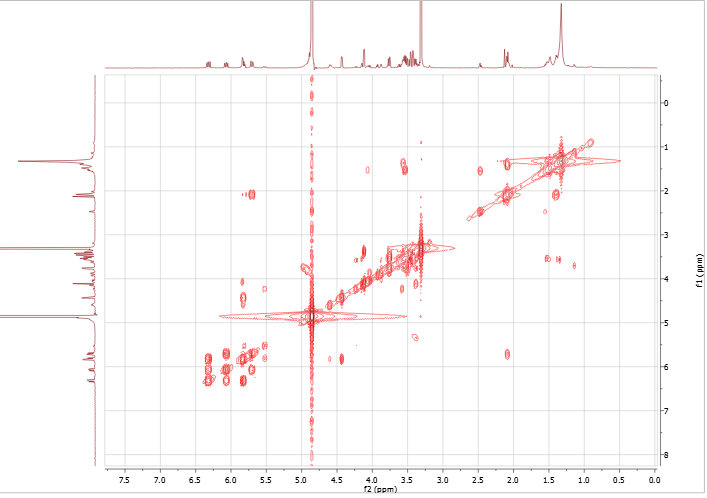


**Figure S35.** HMBC (methanol-*d*_4_) spectrum of compound **5**


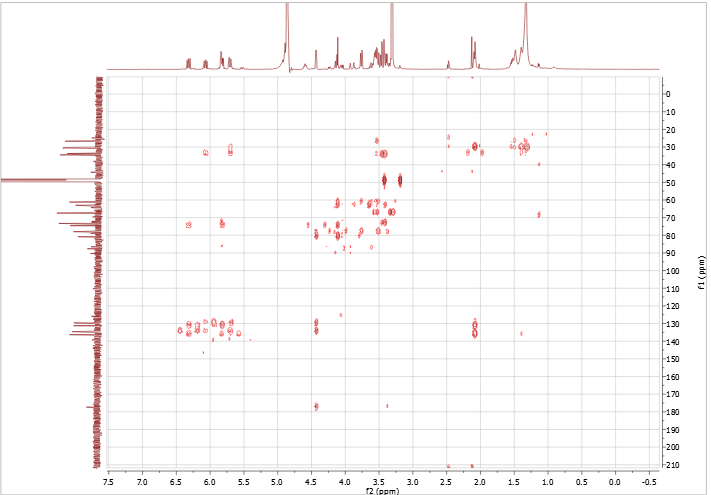


**Figure S36.** ROESY (methanol-*d*_4_) spectrum of compound **5**


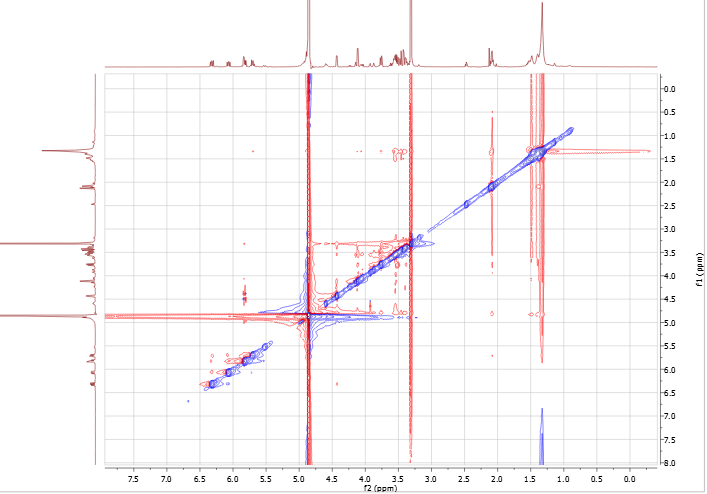


**Figure S37.** HRESIMS of compound **5**


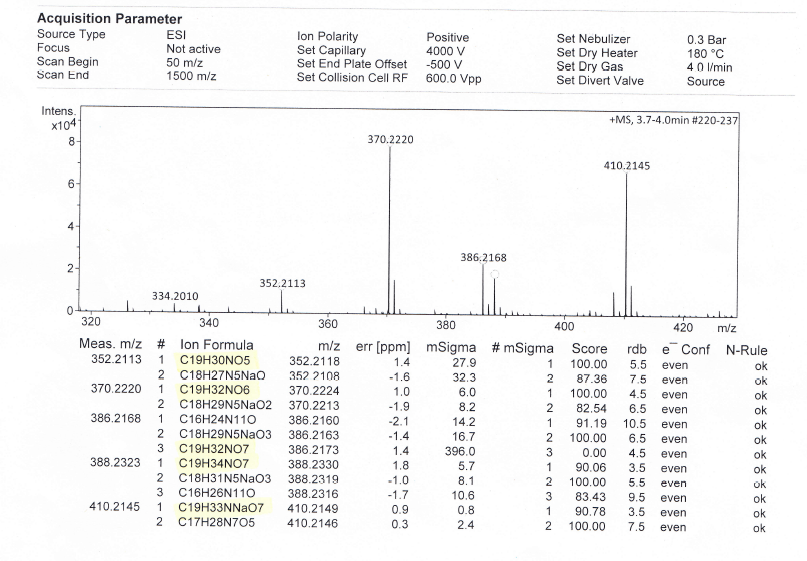


**Figure S38.** HPLC chromatogram of compound **6**

UV absorption of compound **6**

**Figure S39.** ^1^H NMR (600M Hz, methanol-*d*_4_) spectrum of compound **6**


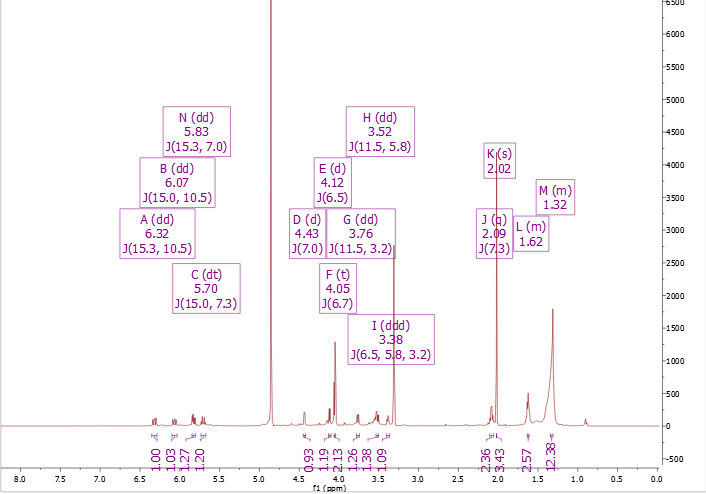


**Figure S40.** ^13^C NMR (150M Hz, methanol-*d*_4_) spectrum of compound **6**


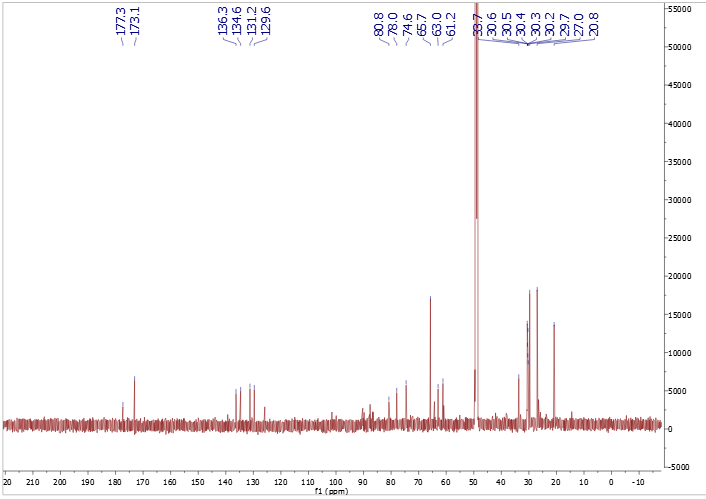


**Figure S41.** HSQC (methanol-*d*_4_) spectrum of compound **6**


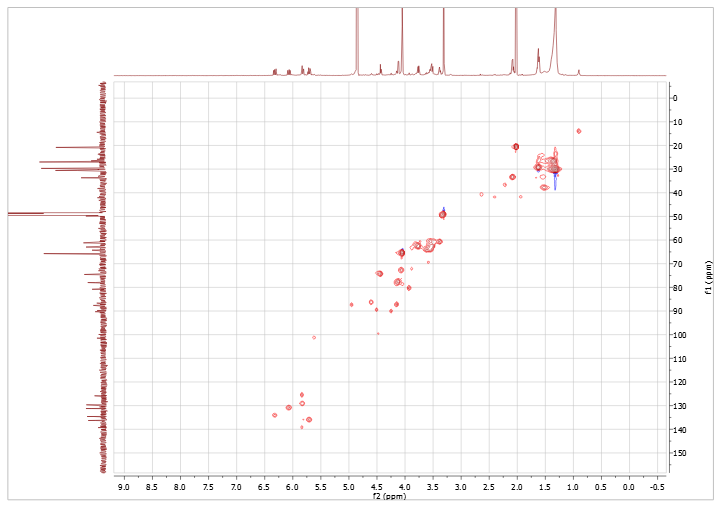


**Figure S42.** COSY (methanol-*d*_4_) spectrum of compound **6**


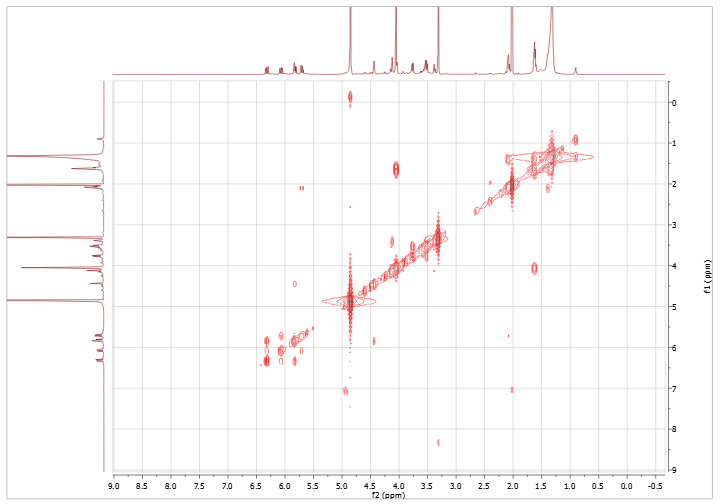


**Figure S43.** HMBC (methanol-*d*_4_) spectrum of compound **6**


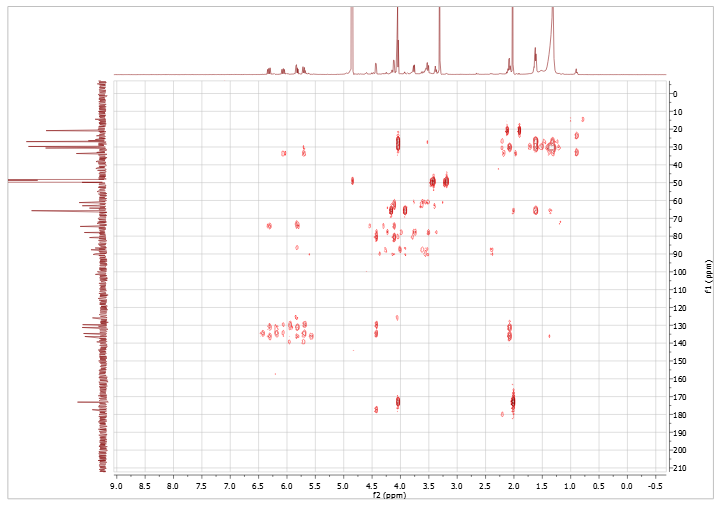


**Figure S44.** ROESY (methanol-*d*_4_) spectrum of compound **6**


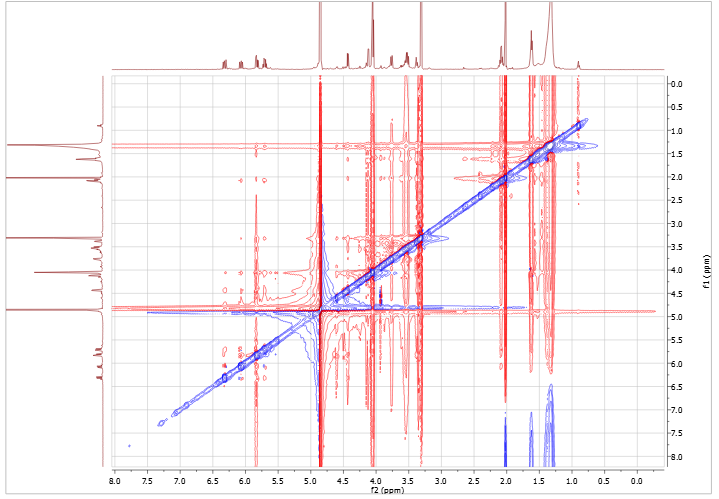


**Figure S45.** HRESIMS of compound **6**


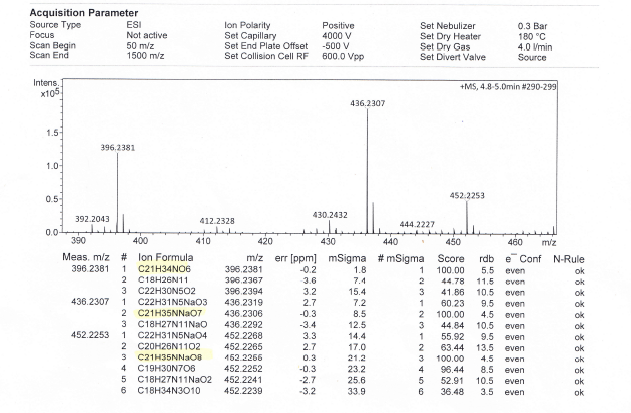


**Figure S46.** HPLC chromatogram of compound **7**

UV absorption of compound **7**

**Figure S47.** ^1^H NMR (600M Hz, methanol-*d*_4_) spectrum of compound **7**


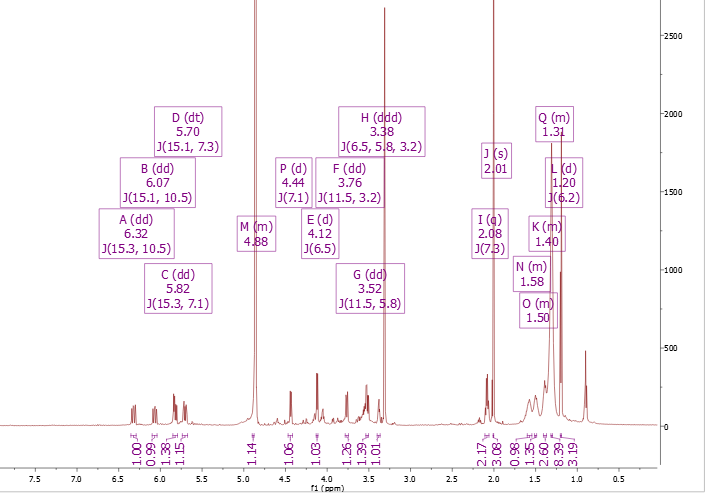


**Figure S48.** ^13^C NMR (150M Hz, methanol-*d*_4_) spectrum of compound **7**


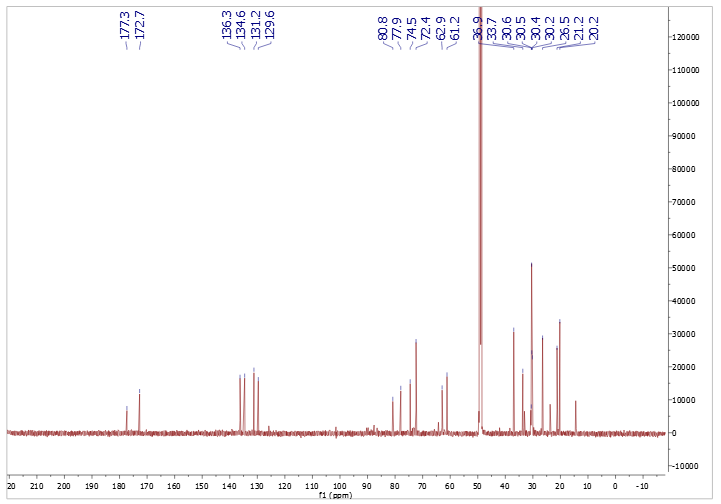


**Figure S49.** HSQC (methanol-*d*_4_) spectrum of compound **7**


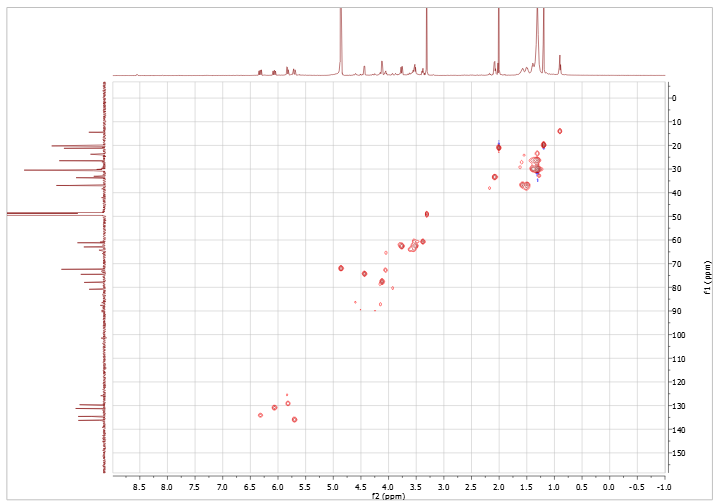


**Figure S50.** COSY (methanol-*d*_4_) spectrum of compound **7**


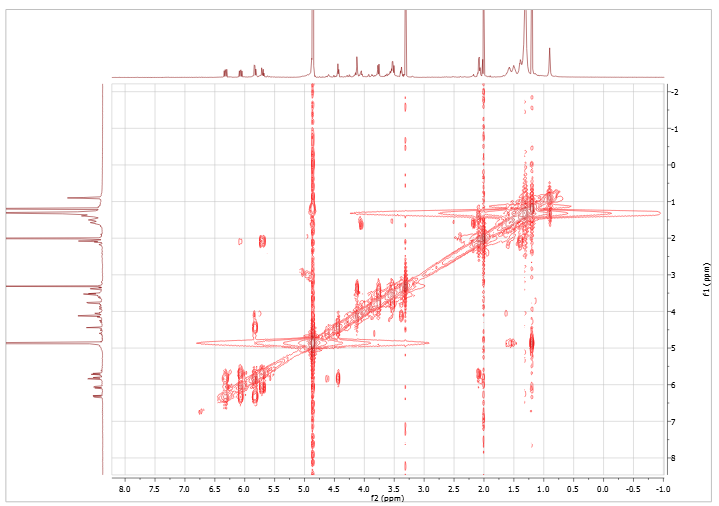


**Figure S51.** HMBC (methanol-*d*_4_) spectrum of compound **7**


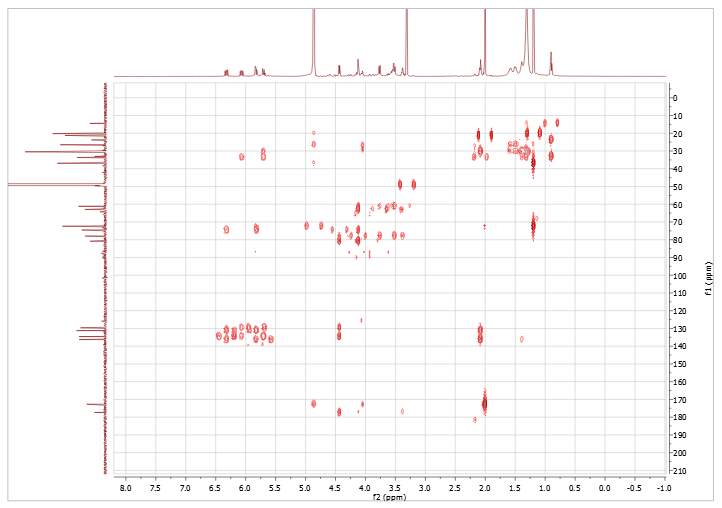


**Figure S52.** ROESY (methanol-*d*_4_) spectrum of compound **7**


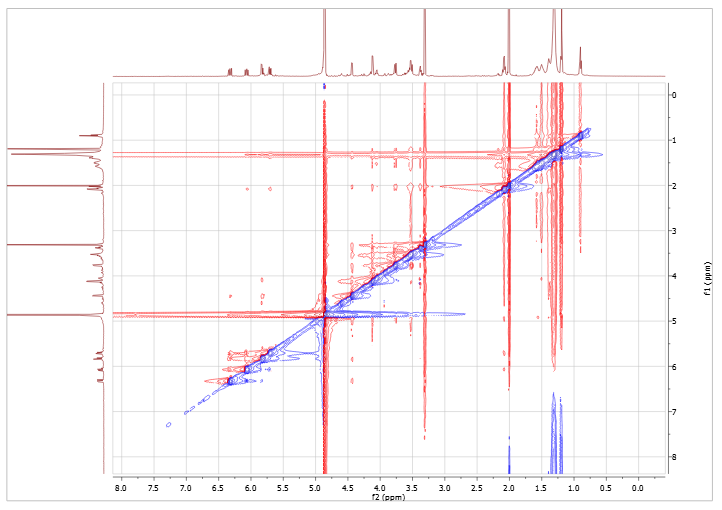


**Figure S53.** HRESIMS of compound **7**


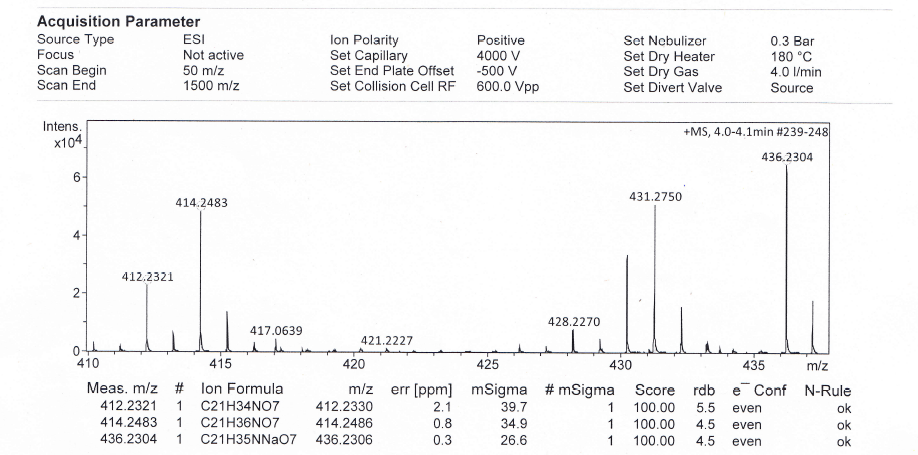


**Figure S54.** HPLC chromatogram of compound **8**

UV absorption of compound **8**

**Figure S55.** ^1^H NMR (600M Hz, methanol-*d*_4_) spectrum of compound **8**


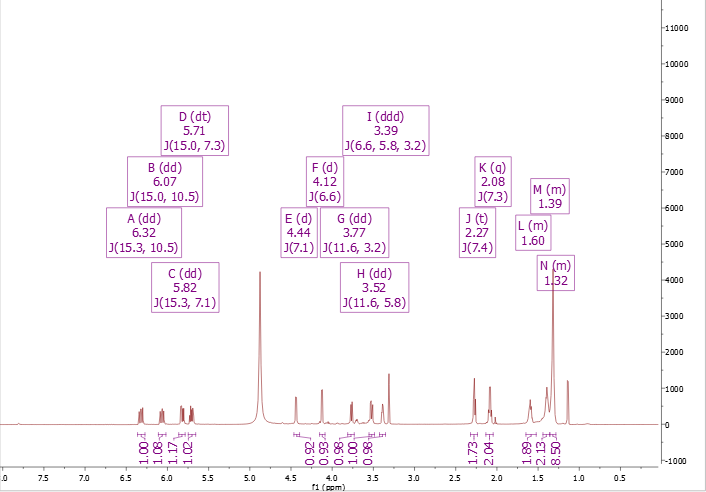


**Figure S56.** ^13^C NMR (150M Hz, methanol-*d*_4_) spectrum of compound **8**


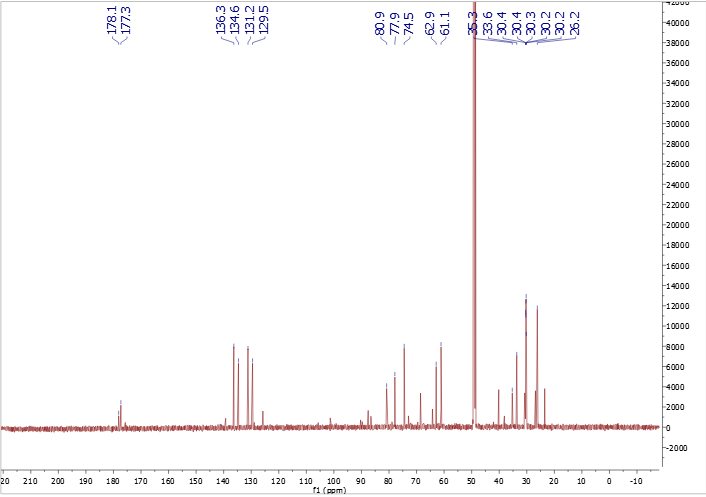


**Figure S57.** HSQC (methanol-*d*_4_) spectrum of compound **8**


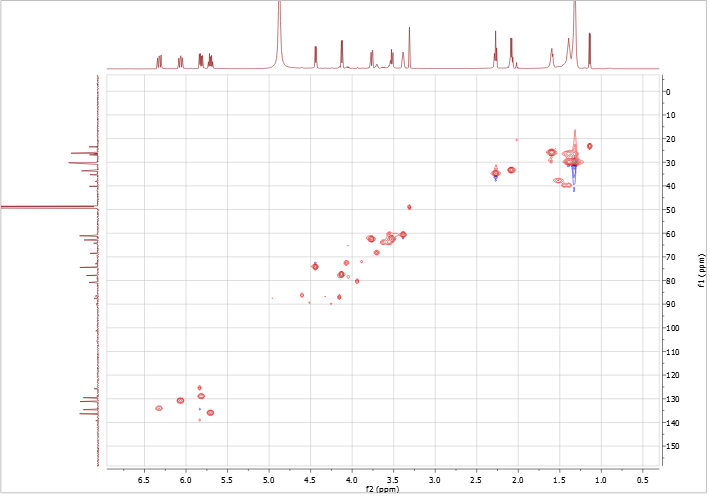


**Figure S58.** COSY (methanol-*d*_4_) spectrum of compound **8**


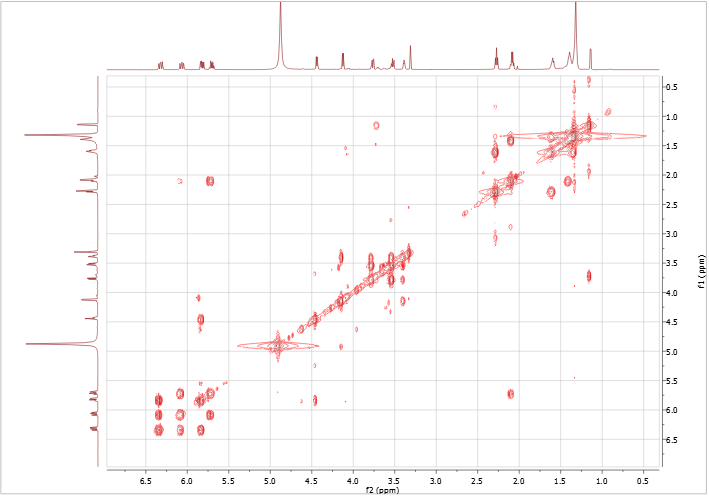


**Figure S59.** HMBC (methanol-*d*_4_) spectrum of compound **8**


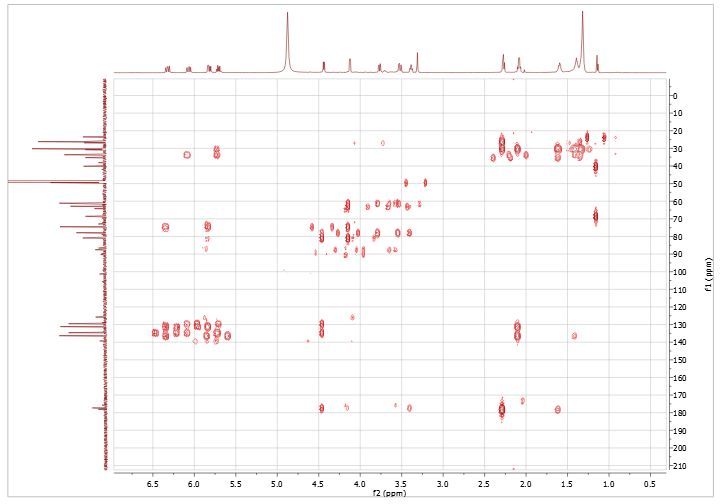


**Figure S60.** ROESY (methanol-*d*_4_) spectrum of compound **8**


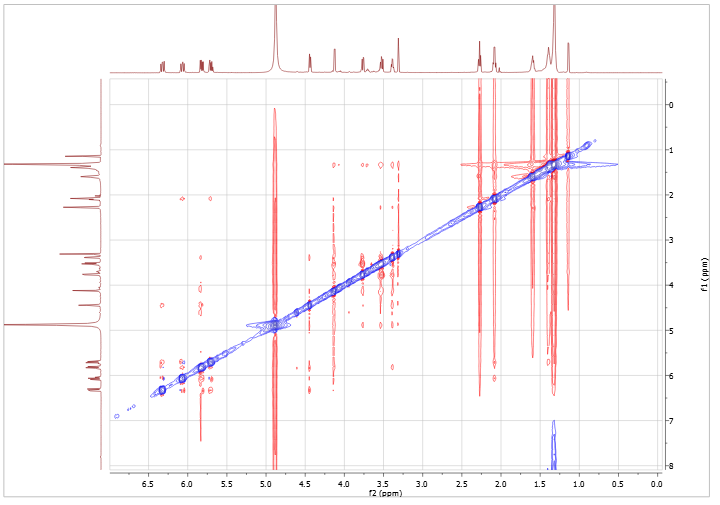


**Figure S61.** HRESIMS of compound **8**


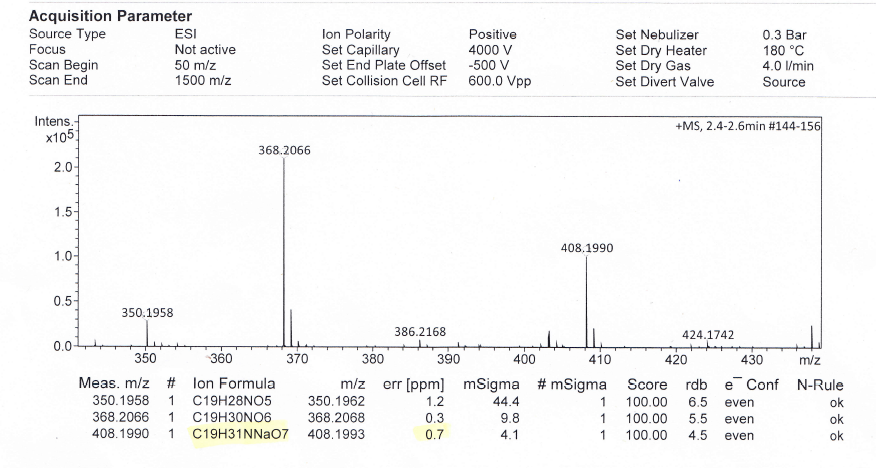


**Figure S62.** HPLC chromatogram of compound **9**

UV absorption of compound **9**

**Figure S63.** ^1^H NMR (600M Hz, methanol-*d*_4_) spectrum of compound **9**


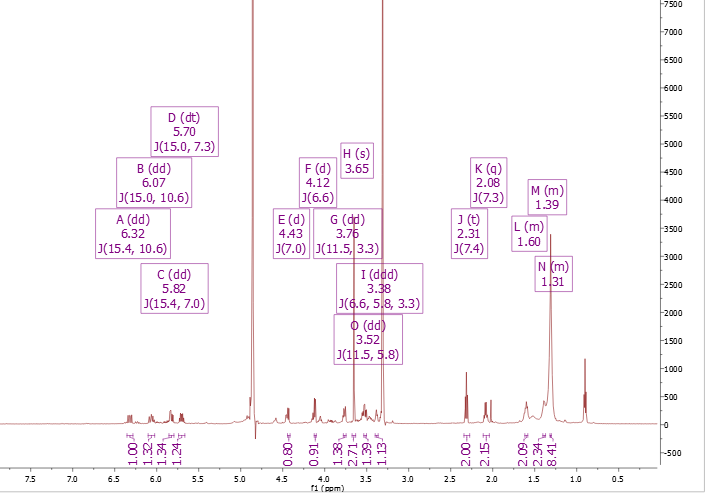


**Figure S64.** ^13^C NMR (150M Hz, methanol-*d*_4_) spectrum of compound **9**


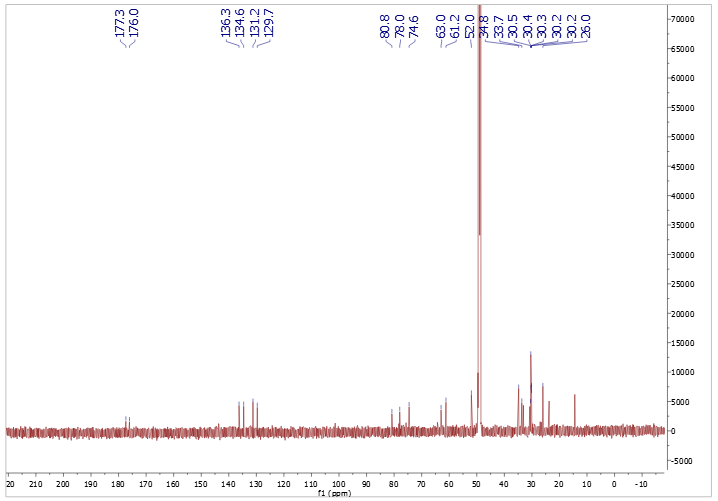


**Figure S65.** HSQC (methanol-*d*_4_) spectrum of compound **9**


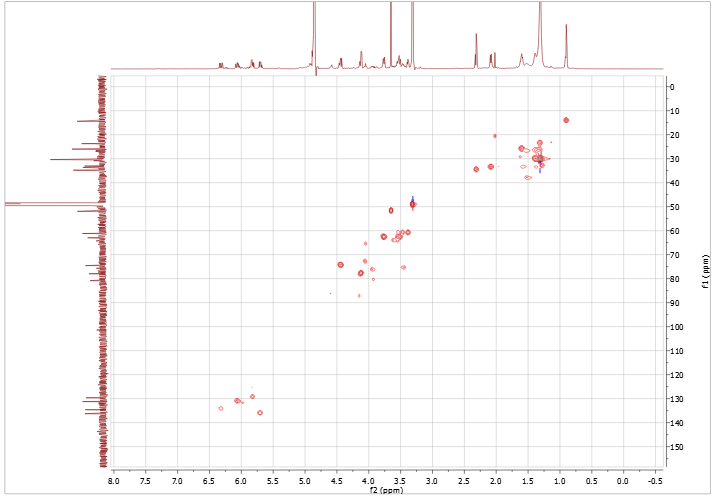


**Figure S66.** COSY (methanol-*d*_4_) spectrum of compound **9**


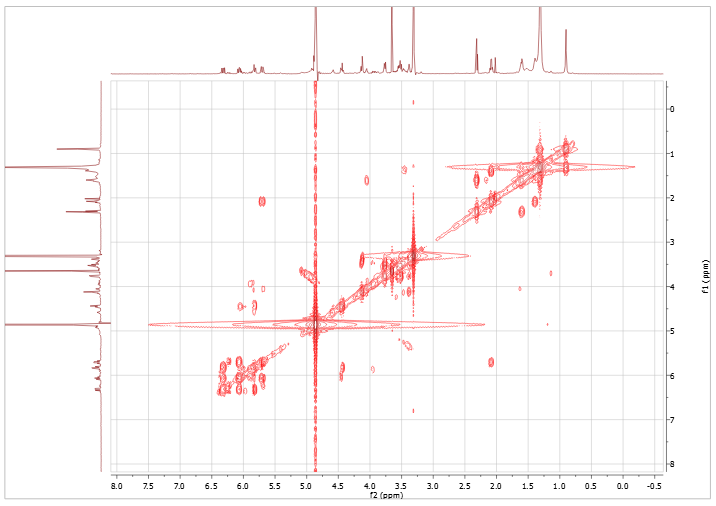


**Figure S67.** HMBC (methanol-*d*_4_) spectrum of compound **9**


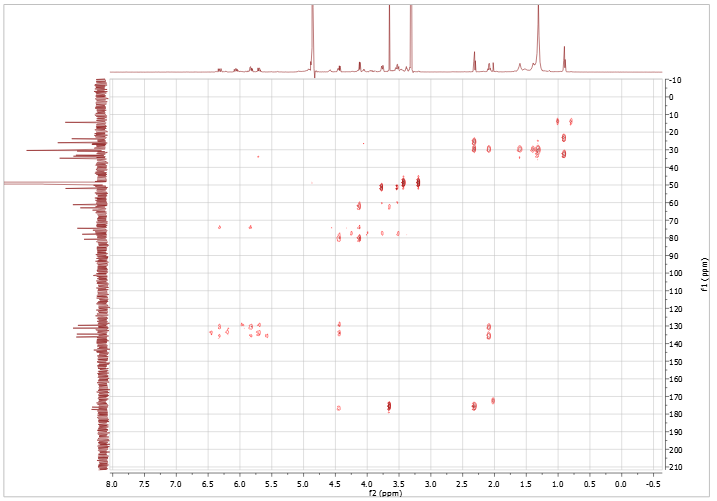


**Figure S68.** ROESY (methanol-*d*_4_) spectrum of compound **9**


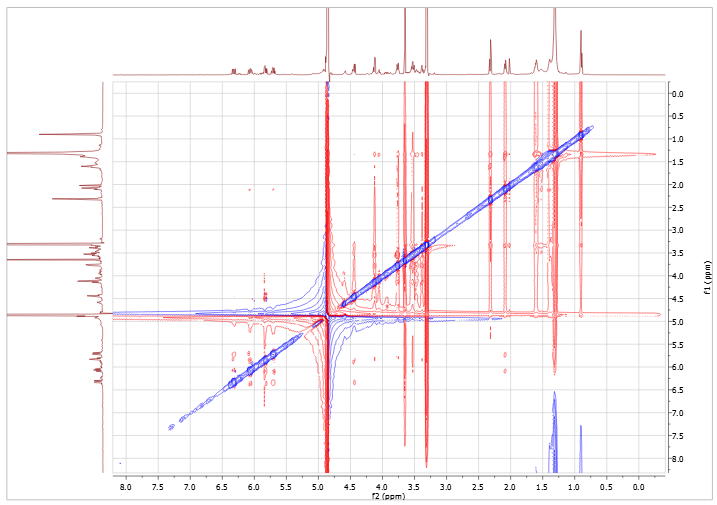


**Figure S69.** HRESIMS of compound **9**


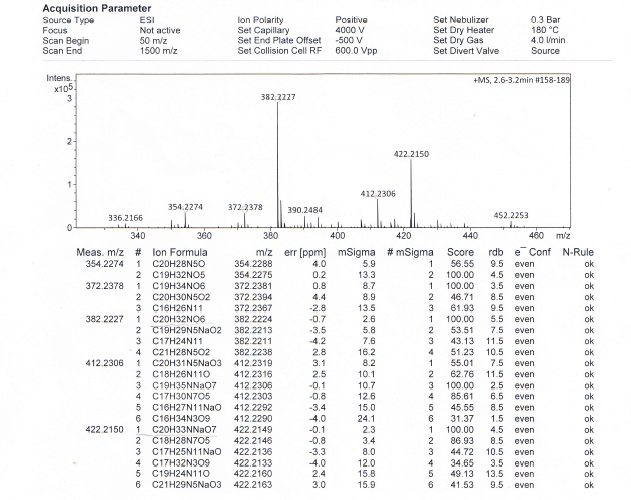


**Figure S70.** HPLC chromatogram of compound **10**

UV absorption of compound **10**

**Figure S71.** ^1^H NMR (600M Hz, methanol-*d*_4_) spectrum of compound **10**


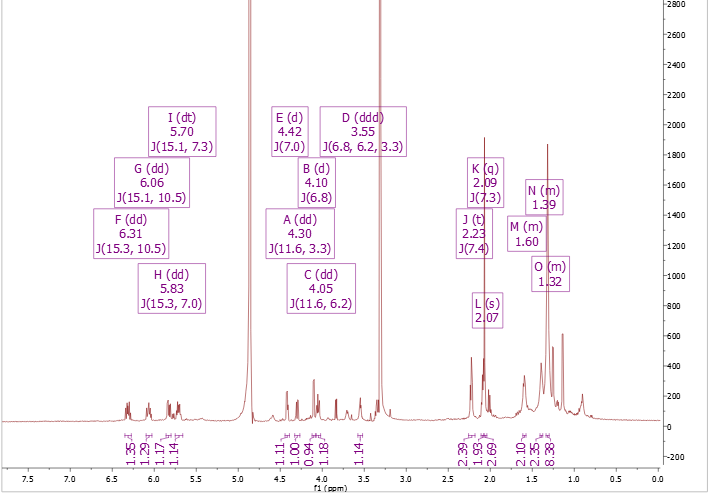


**Figure S72.** HSQC (methanol-*d*_4_) spectrum of compound **10**


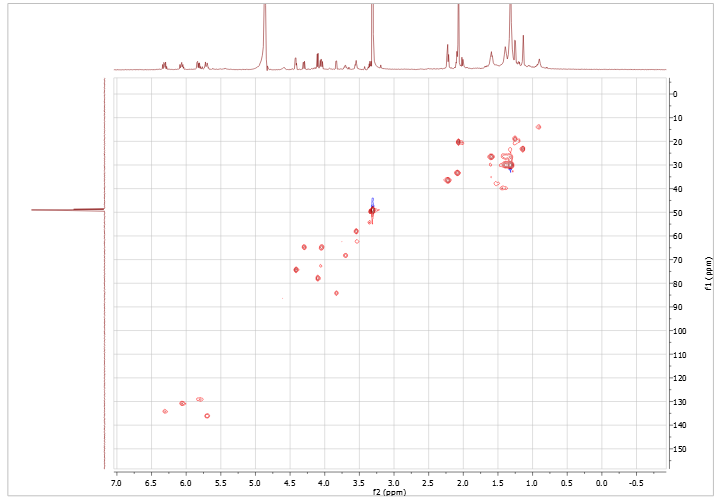


**Figure S73.** COSY (methanol-*d*_4_) spectrum of compound **10**


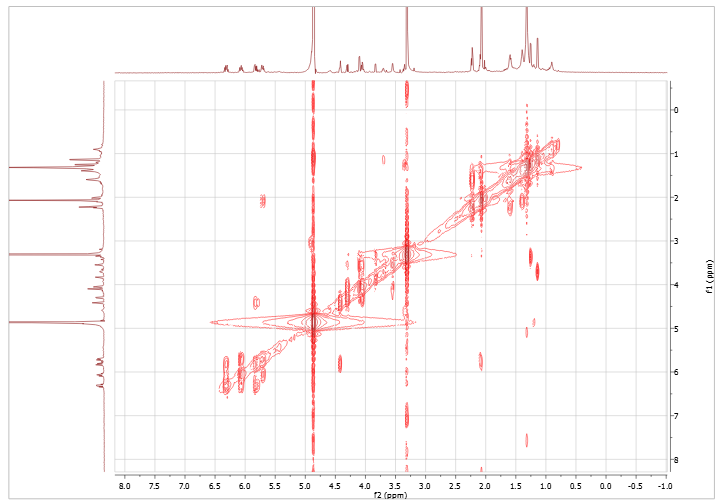


**Figure S74.** HMBC (methanol-*d*_4_) spectrum of compound **10**


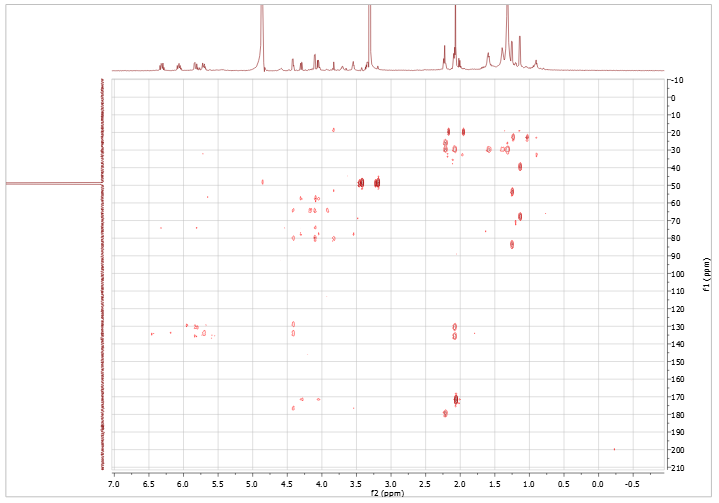


**Figure S75.** ROESY (methanol-*d*_4_) spectrum of compound **10**


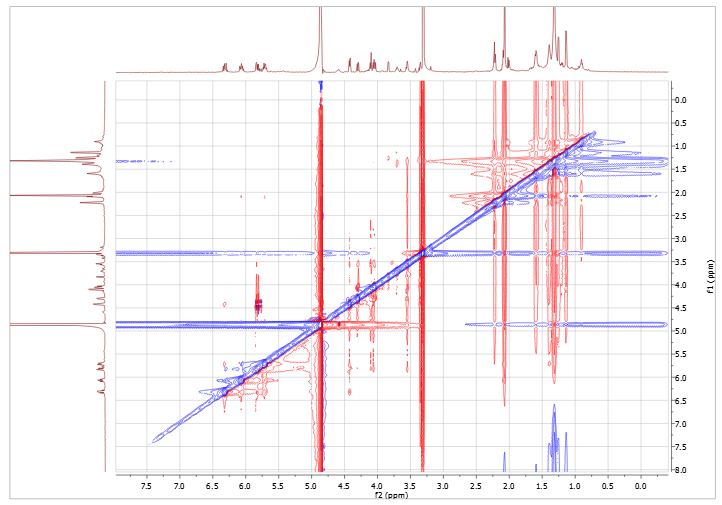


**Figure S76.** HRESIMS of compound **10**


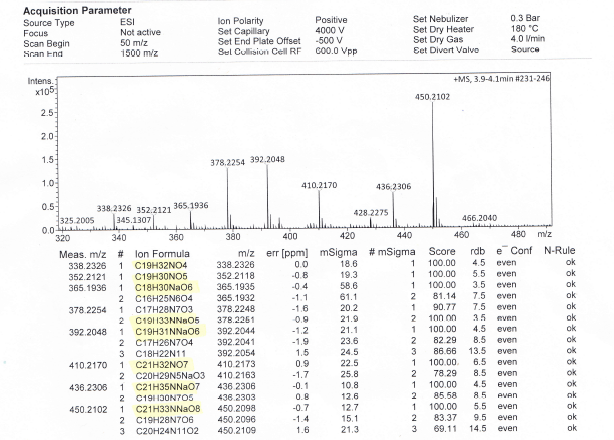


**Figure S77.** HPLC chromatogram of compound **11**

UV absorption of compound **11**

**Figure S78.** ^1^H NMR (600M Hz, methanol-*d*_4_) spectrum of compound **11**


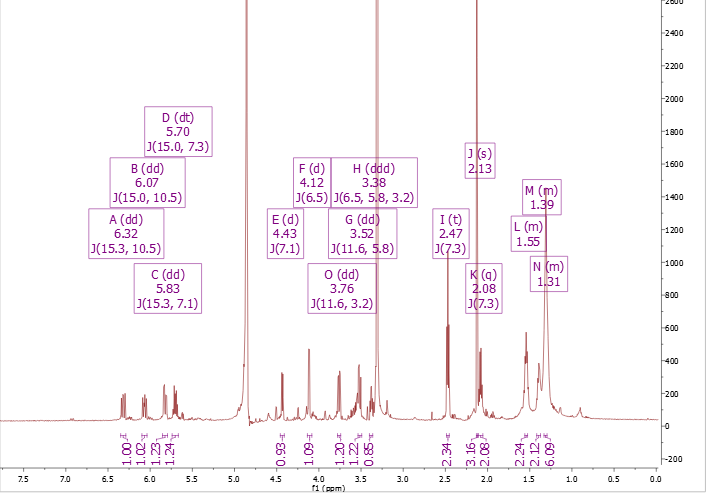


**Figure S79.** ^13^C NMR (150M Hz, methanol-*d*_4_) spectrum of compound **11**


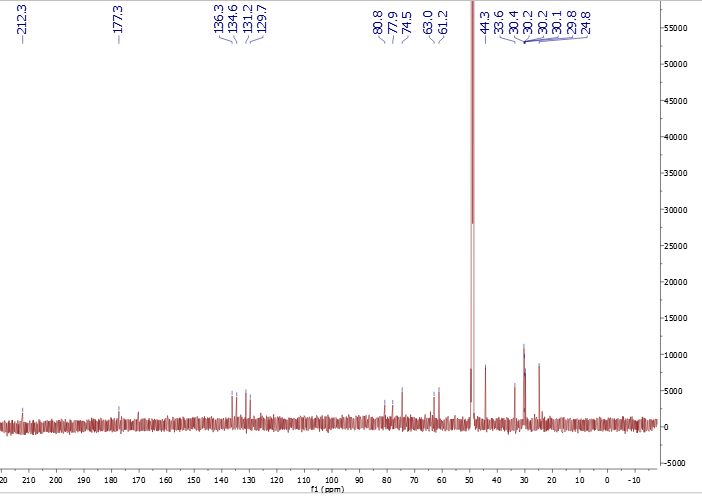


**Figure S80.** HSQC (methanol-*d*_4_) spectrum of compound **11**


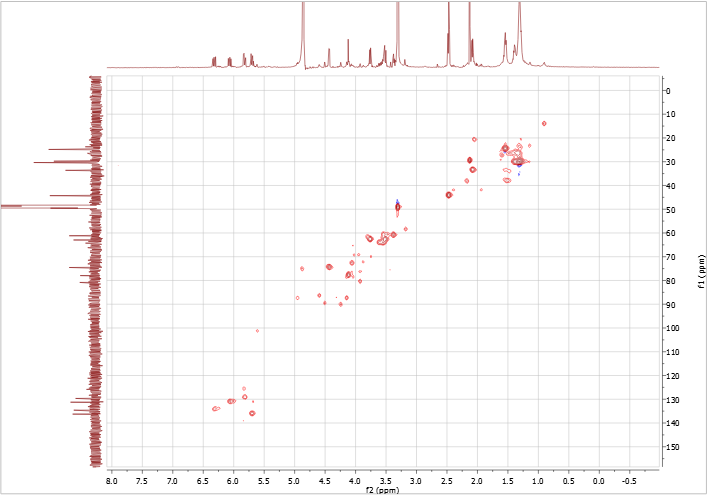


**Figure S81.** COSY (methanol-*d*_4_) spectrum of compound **11**


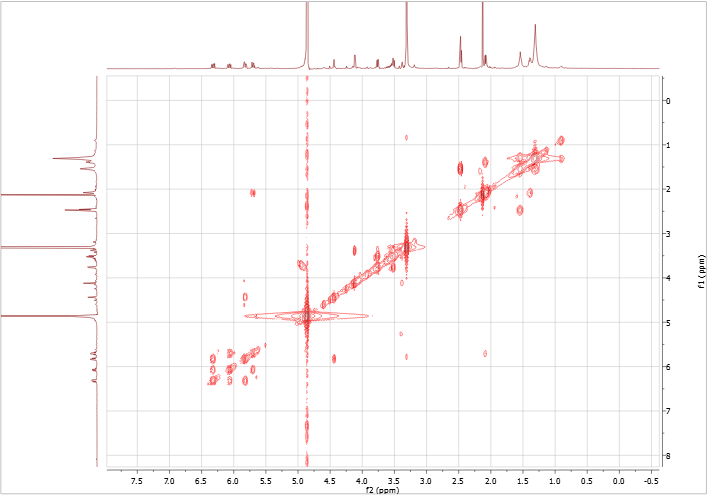


**Figure S82.** HMBC (methanol-*d*_4_) spectrum of compound **11**


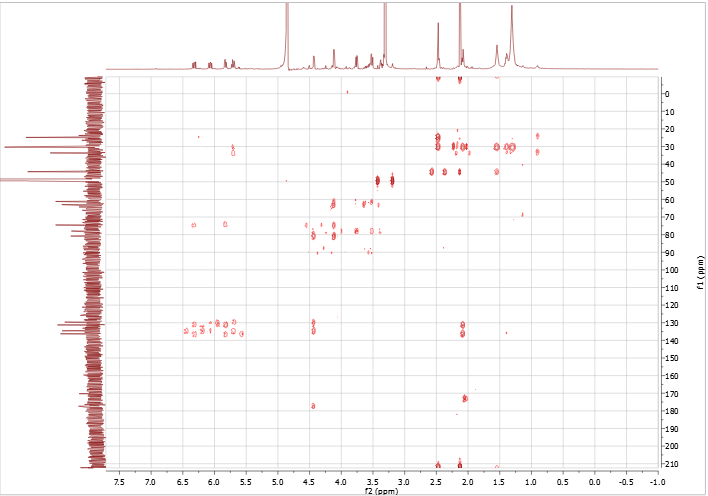


**Figure S83.** ROESY (methanol-*d*_4_) spectrum of compound **11**


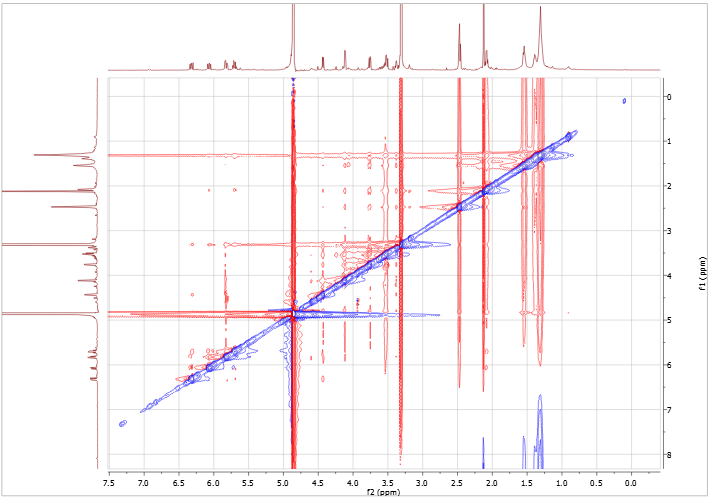


**Figure S84.** HRESIMS of compound **11**


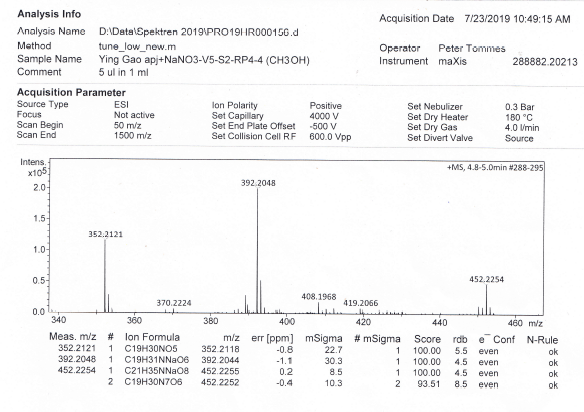


**Figure S85.** HPLC chromatogram of compound **12**

UV absorption of compound **12**

**Figure S86.** ^1^H NMR (600M Hz, methanol-*d*_4_) spectrum of compound **12**


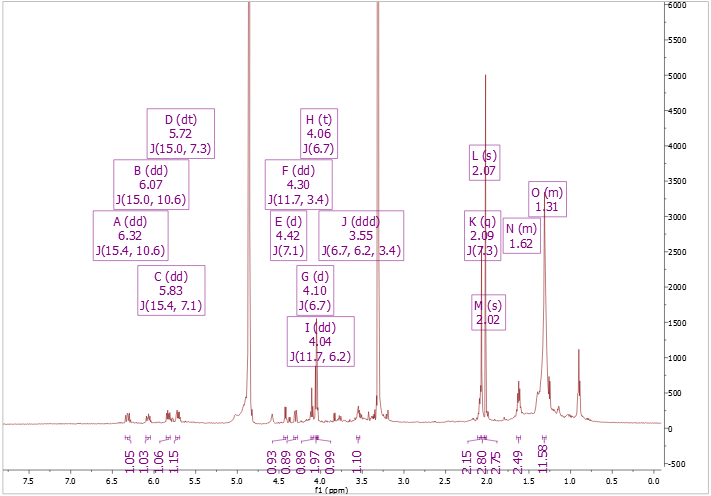


**Figure S87.** HSQC (methanol-*d*_4_) spectrum of compound **12**


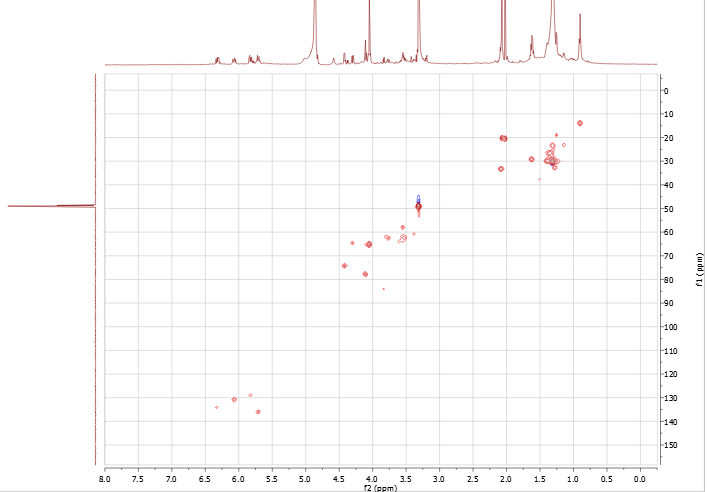


**Figure S88.** COSY (methanol-*d*_4_) spectrum of compound **12**


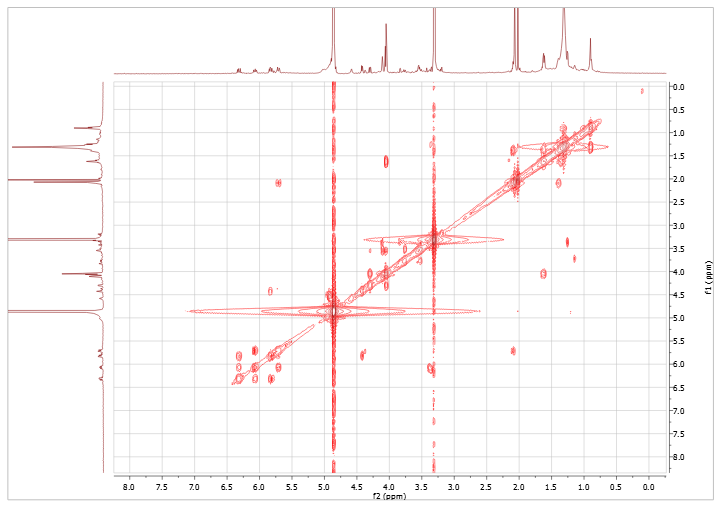


**Figure S89.** HMBC (methanol-*d*_4_) spectrum of compound **12**


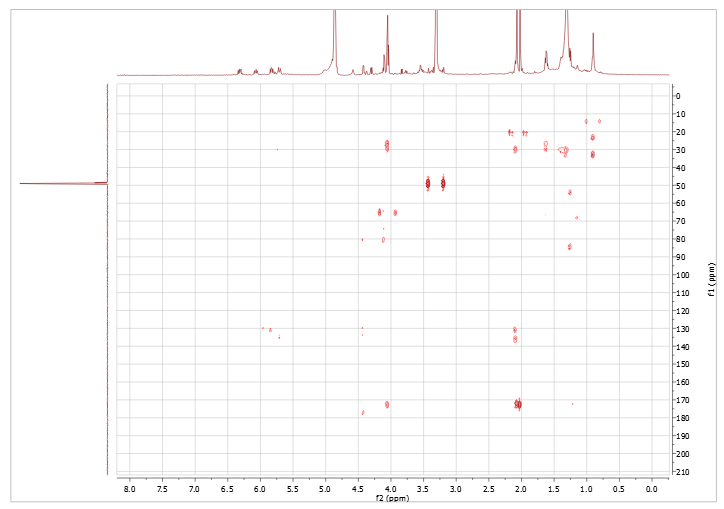


**Figure S90.** ROESY (methanol-*d*_4_) spectrum of compound **12**


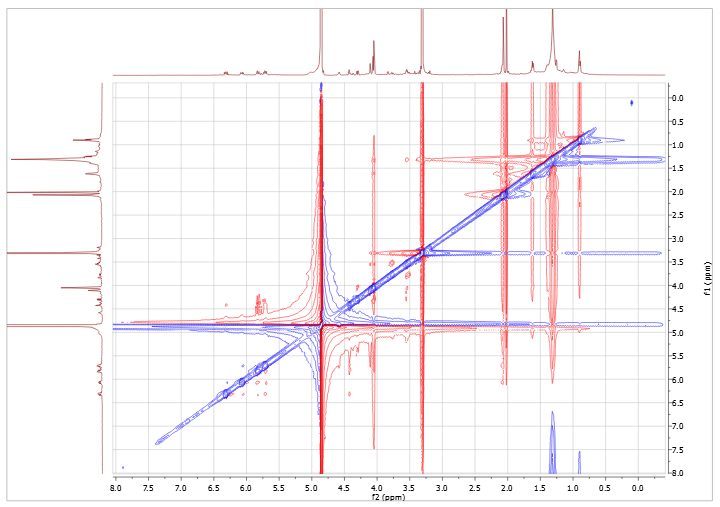


**Figure S91.** HRESIMS of compound **12**


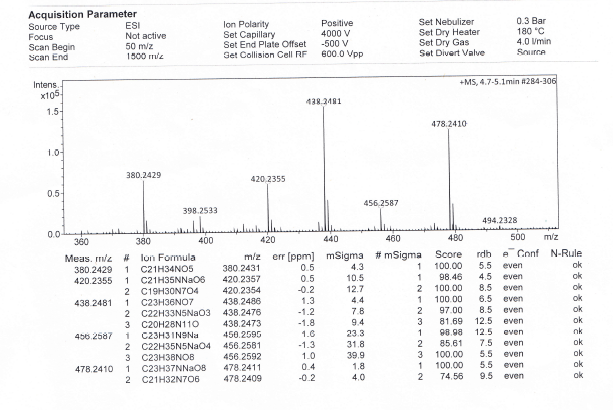


**Figure S92.** Low-energy conformers and populations of (3*S*,4*S*,5*S*)-**1mod** computed at the ωB97X/TZVP PCM/MeOH level of theory.


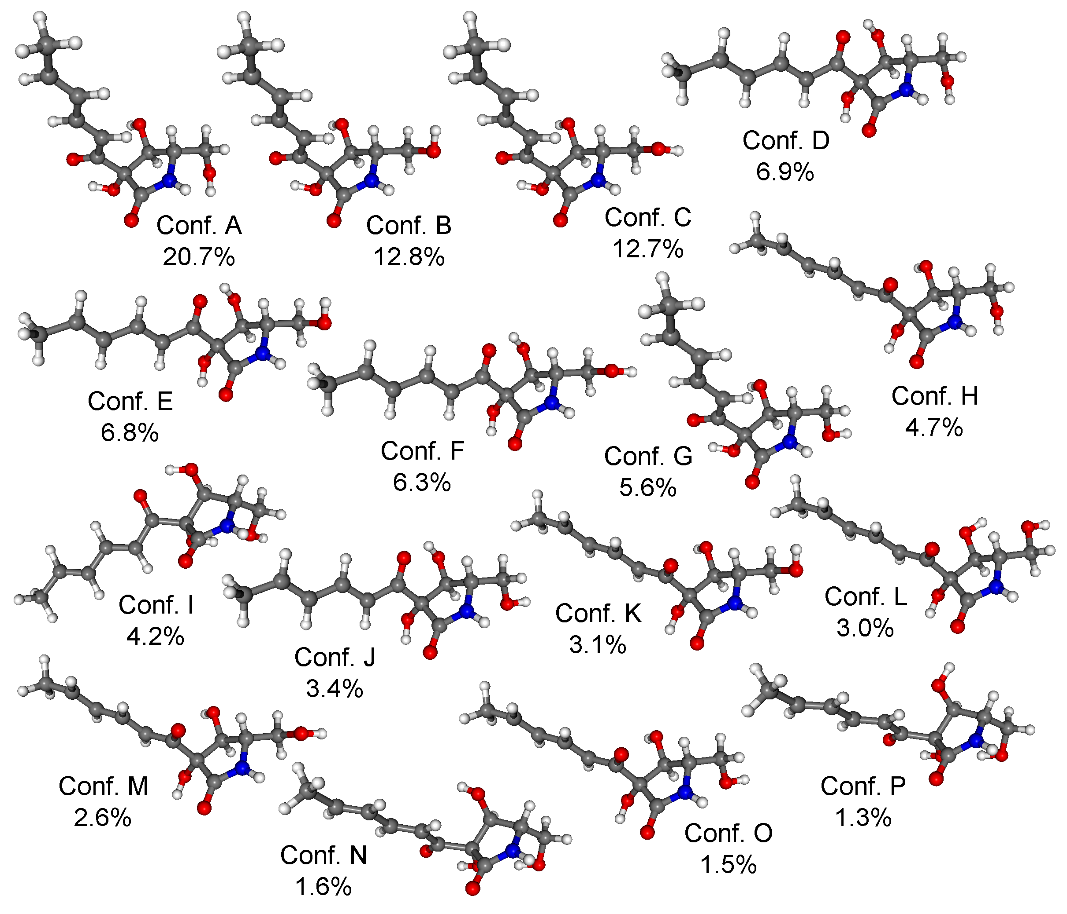


**Figure S93.** Low-energy conformers and populations of (3*R*,4*S*,5*S*,7*R*)-**2mod** computed at the B3LYP/6-31+G(d,p) level of theory.


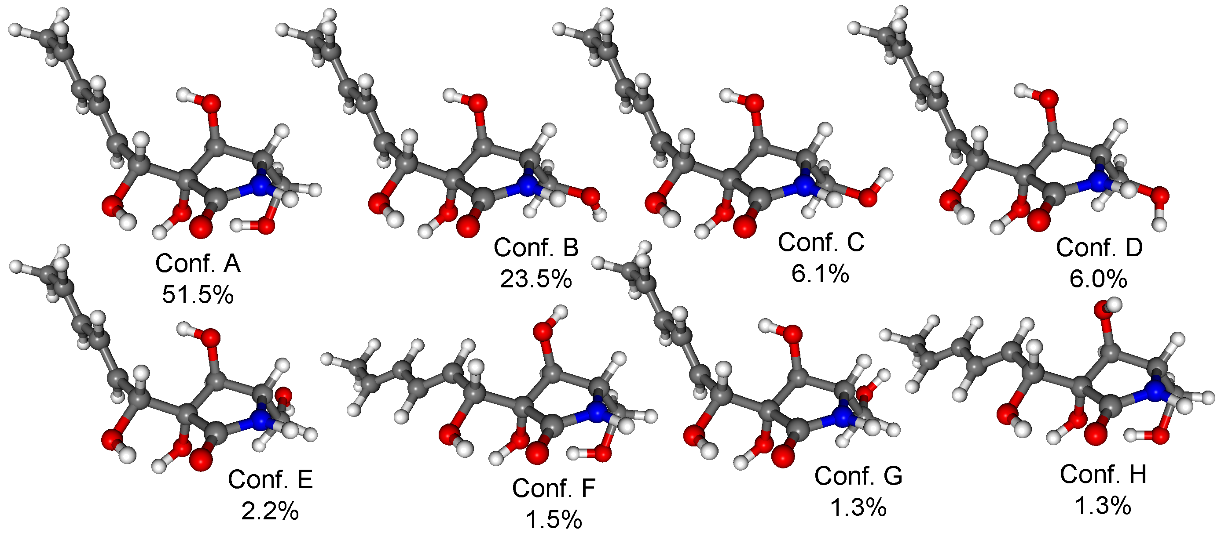


**Figure S94.** Low-energy conformers and populations of (3*R*,4*S*,5*S*,7*S*)-**2mod** computed at the B3LYP/6-31+G(d,p) level of theory.


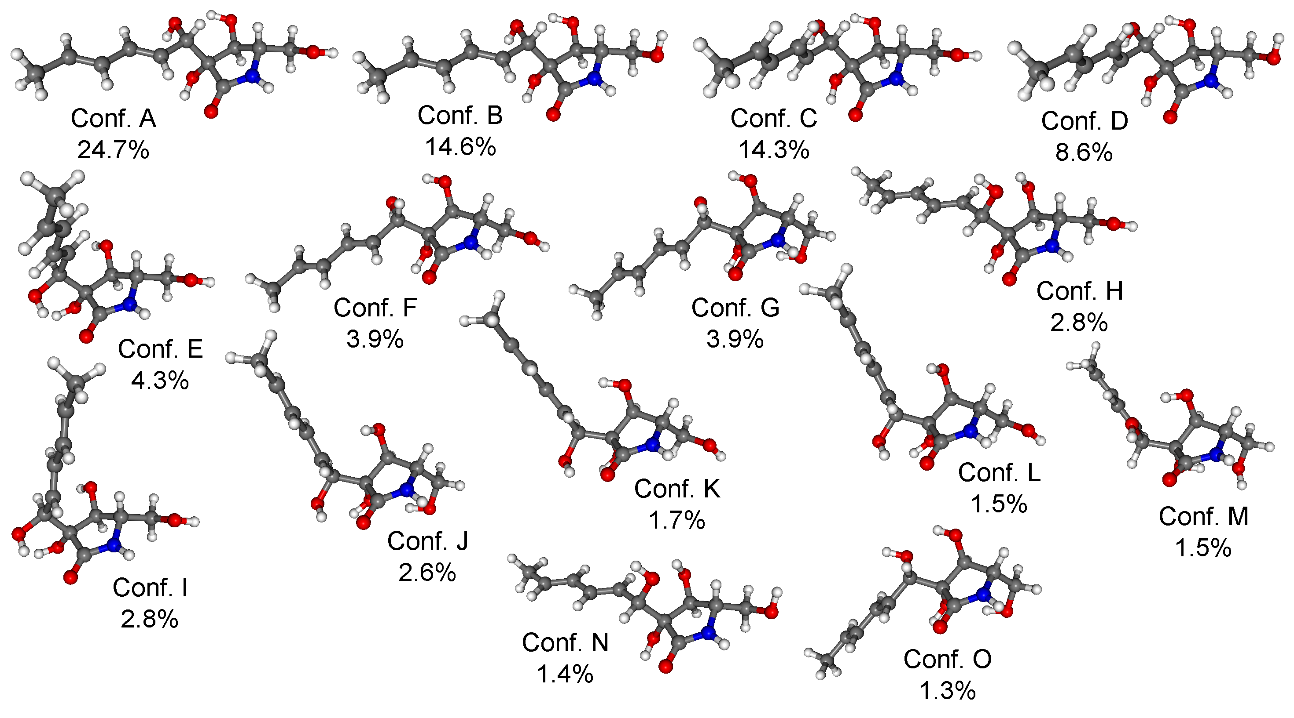


**Figure S95.** Experimental ECD spectrum of **2** in MeCN compared with the Boltzmann-weighted B3LYP/TZVP PCM/MeCN ECD spectrum of (3*R*,4*S*,5*S*,7*R*)-**2mod**.

Level of optimization: ωB97X/TZVP PCM/MeCN. Bars represent the rotatory strength values of the lowest-energy conformer. The experimental spectrum was scaled to the computed one.


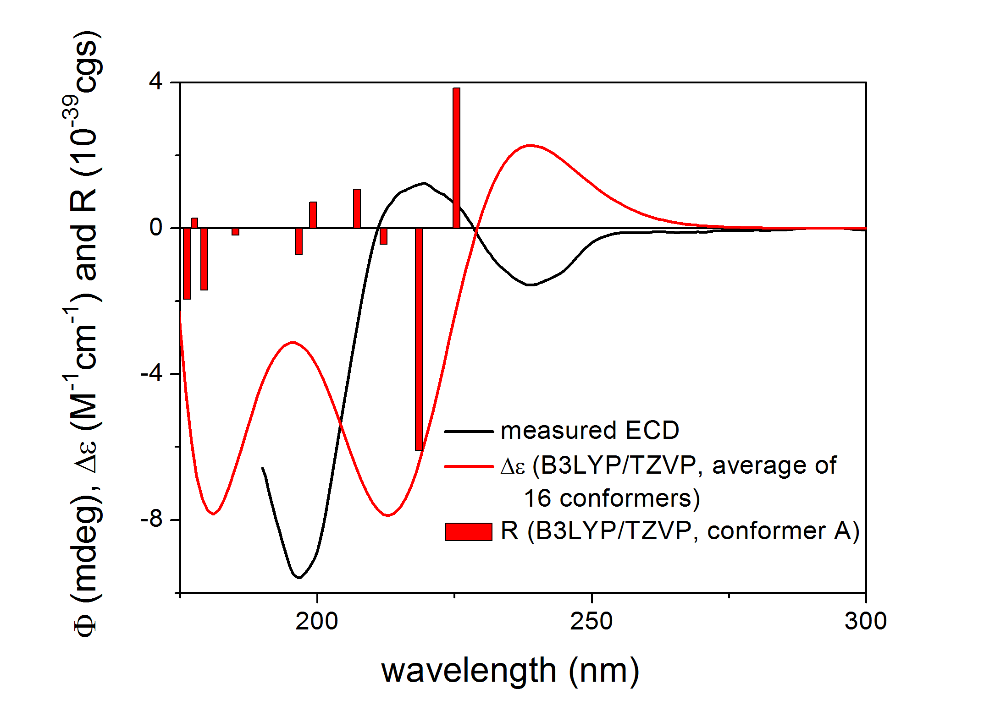


**Figure S96.** Low-energy conformers and populations of (3*R*,4*S*,5*S*,7*R*)-**2mod** computed at the ωB97X/TZVP PCM/MeCN level of theory.


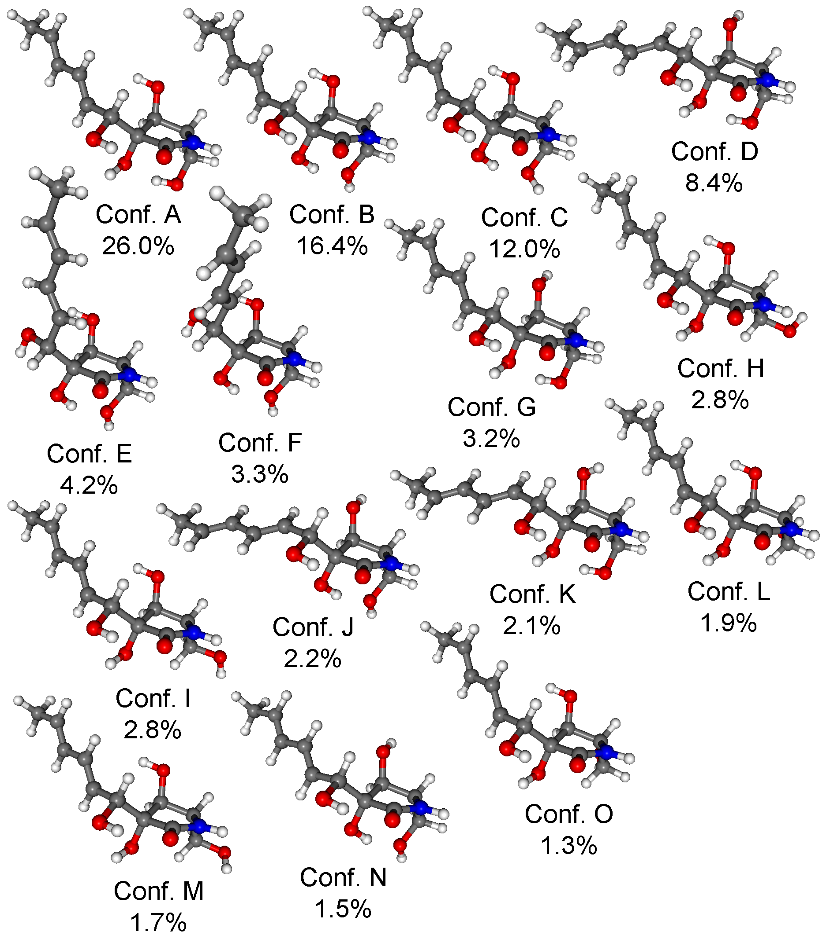


**Figure S97.** Low-energy conformers and populations of (3*R*,4*S*,5*S*,7*S*)-**2mod** computed at the ωB97X/TZVP PCM/MeCN level of theory.


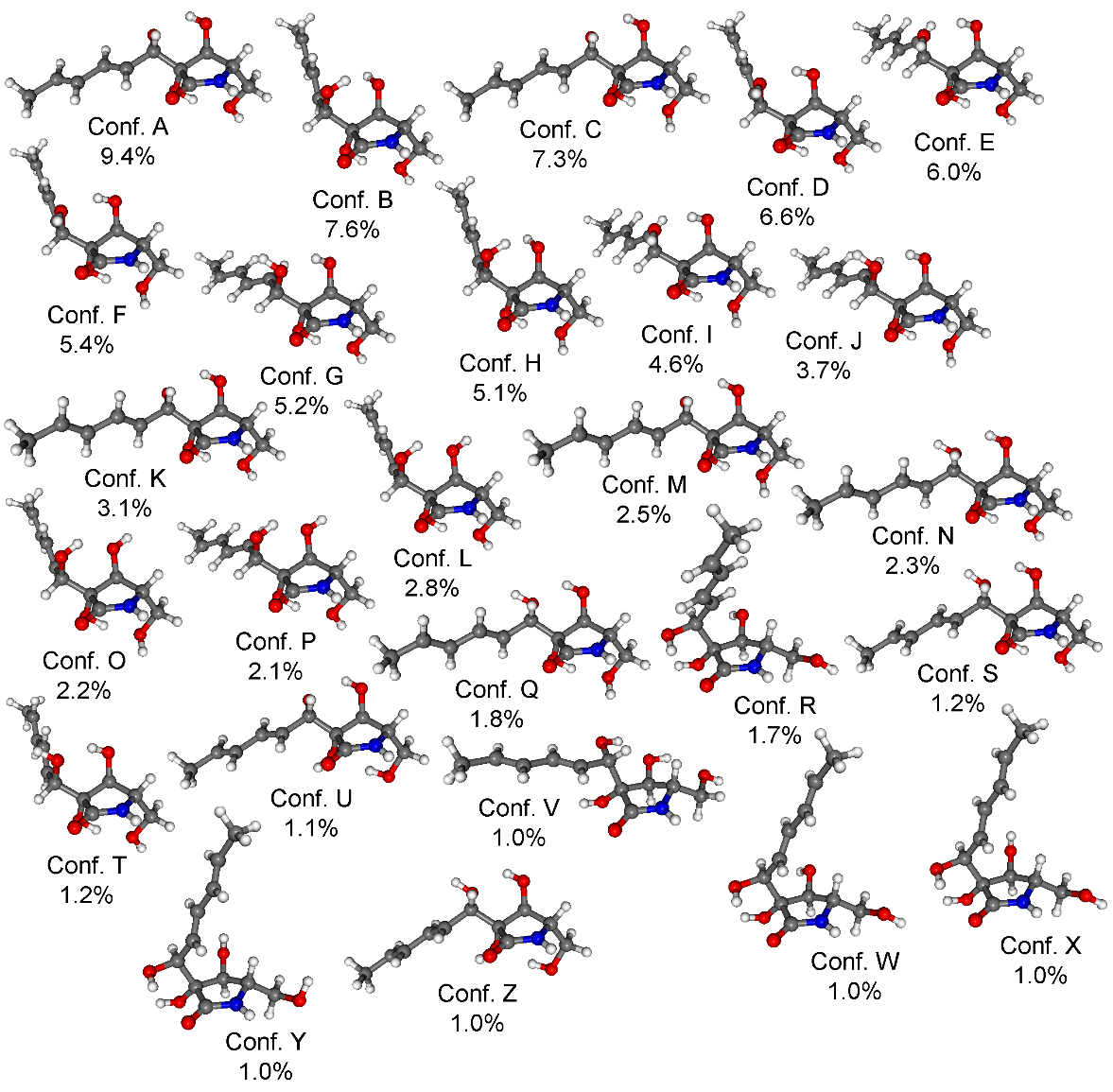


**Table S1.** Computed SOR values for (3*S*,4*S*,5*S*)-**1mod** at various levels.

| Level of optimization | Boltzmann population (%) | ωB97X/TZVP PCM/MeOH | | | |
| --- | --- | --- | --- | --- | --- |
| Level of OR calculation |  | B3LYP/TZVP PCM/MeOH | BH&HLYP/TZVP PCM/MeOH | CAM-B3LYP/TZVP PCM/MeOH | PBE0/TZVP PCM/MeOH |
| Conf A | 20.73 | -60.03 | -51.48 | -58.60 | -57.66 |
| Conf B | 12.79 | -64.09 | -64.99 | -70.75 | -62.59 |
| Conf C | 12.68 | -49.89 | -51.60 | -56.93 | -49.38 |
| Conf D | 6.88 | -318.03 | -212.93 | -237.03 | -295.78 |
| Conf E | 6.84 | -339.21 | -239.35 | -263.70 | -317.02 |
| Conf F | 6.34 | -324.85 | -225.96 | -249.83 | -303.68 |
| Conf G | 5.63 | -70.64 | -65.52 | -72.08 | -69.20 |
| Conf H | 4.74 | -70.11 | -65.06 | -62.20 | -64.43 |
| Conf I | 4.24 | 58.63 | 40.13 | 49.06 | 55.72 |
| Conf J | 3.41 | -318.35 | -215.63 | -239.63 | -296.42 |
| Conf K | 3.11 | -98.38 | -100.36 | -97.64 | -93.64 |
| Conf L | 3.02 | 54.80 | 31.22 | 43.09 | 57.26 |
| Conf M | 2.55 | -88.36 | -90.06 | -86.62 | -84.33 |
| Conf N | 1.60 | 69.08 | 50.40 | 51.91 | 74.14 |
| Conf O | 1.48 | -71.23 | -72.71 | -68.89 | -66.53 |
| Conf P | 1.31 | -11.95 | -17.24 | -16.59 | -11.74 |
| BW Average |  | -115.13 | -90.67 | -98.66 | -108.36 |

**Table S2.** Comparison of the experimental ^13^C NMR data of the carbons of the **2** measured in MeOH-*d*_4_ with the mPW1PW91/6-311+G(2d,p) // B3LYP/6-31+G(d,p) ones of the (3*R*,4*S*,5*S*,7*R*)-**2mod** and (3*R*,4*S*,5*S*,7*S*)-**2mod** stereoisomers.

| Numbering | Exp | Calc_SSSR_ | Calc_SSSS_ | Δδ_SSSR_ | Δδ_SSSS_ |
| --- | --- | --- | --- | --- | --- |
| C-2 | 177.3 | 179.49 | 174.87 | 2.19 | 2.43 |
| C-3 | 80.8 | 76.60 | 80.15 | 4.20 | 0.65 |
| C-4 | 77.9 | 78.39 | 80.71 | 0.49 | 2.81 |
| C-5 | 61.2 | 63.70 | 61.33 | 2.50 | 0.13 |
| C-6 | 62.9 | 64.58 | 66.51 | 1.68 | 3.61 |
| C-7 | 74.5 | 74.63 | 74.88 | 0.13 | 0.38 |
| C-8 | 129.6 | 130.54 | 130.27 | 0.94 | 0.67 |
| C-9 | 134.6 | 139.14 | 137.72 | 4.54 | 3.12 |
| C-10 | 131.2 | 133.58 | 134.07 | 2.38 | 2.87 |
| C-11 | 136.3 | 138.92 | 135.96 | 2.62 | 0.34 |
| C-12 | 33.7 | 19.51 | 19.47 | 14.19 | 14.23 |
| Average | N/A | N/A | N/A | 3.26 | 2.84 |
| Average without C-12 | N/A | N/A | N/A | 2.17 | 1.70 |

**Table S3.** Computed SOR values for (3*R*,4*S*,5*S*,7*R*)-**2mod** at various levels.

| Level of optimization | Boltzmann population (%) | ωB97X/TZVP PCM/MeOH | | | |
| --- | --- | --- | --- | --- | --- |
| Level of OR calculation |  | B3LYP/TZVP PCM/MeOH | BH&HLYP/TZVP PCM/MeOH | CAM-B3LYP/TZVP PCM/MeOH | PBE0/TZVP PCM/MeOH |
| Conf A | 26.54 | -31.08 | -19.24 | -20.12 | -30.61 |
| Conf B | 16.04 | -45.15 | -36.95 | -37.62 | -43.31 |
| Conf C | 11.70 | -53.17 | -40.90 | -42.15 | -51.37 |
| Conf D | 8.47 | 214.61 | 175.58 | 186.10 | 207.09 |
| Conf E | 4.21 | 193.76 | 164.87 | 172.43 | 191.92 |
| Conf F | 3.24 | -28.52 | -27.21 | -24.12 | -30.76 |
| Conf G | 3.21 | -65.79 | -55.78 | -55.78 | -61.12 |
| Conf H | 2.89 | -103.64 | -85.77 | -90.43 | -102.12 |
| Conf I | 2.85 | -83.68 | -70.92 | -73.96 | -83.06 |
| Conf J | 2.14 | 187.81 | 159.58 | 165.90 | 184.01 |
| Conf K | 2.06 | 208.76 | 176.86 | 183.19 | 203.53 |
| Conf L | 1.90 | 34.89 | 27.74 | 32.44 | 32.81 |
| Conf M | 1.76 | -63.37 | -48.68 | -51.64 | -63.37 |
| Conf N | 1.44 | -29.30 | -33.19 | -29.44 | -30.24 |
| Conf O | 1.29 | 6.38 | 4.40 | 9.01 | 6.30 |
| BW Average |  | 4.18 | 5.73 | 6.87 | 3.97 |

**Table S4.** Computed SOR values for (3*R*,4*S*,5*S*,7*S*)-**2mod** at various levels.

| Level of optimization | Boltzmann population (%) | ωB97X/TZVP PCM/MeOH | | | |
| --- | --- | --- | --- | --- | --- |
| Level of OR calculation |  | B3LYP/TZVP PCM/MeOH | BH&HLYP/TZVP PCM/MeOH | CAM-B3LYP/TZVP PCM/MeOH | PBE0/TZVP PCM/MeOH |
| Conf A | 9.40 | -84.26 | -75.84 | -70.66 | -85.35 |
| Conf B | 7.44 | -64.78 | -61.18 | -57.65 | -64.12 |
| Conf C | 7.36 | -101.23 | -88.55 | -83.34 | -102.01 |
| Conf D | 6.68 | -84.84 | -77.74 | -77.61 | -85.62 |
| Conf E | 6.06 | 172.44 | 139.47 | 148.03 | 164.32 |
| Conf F | 5.47 | -93.73 | -83.26 | -83.85 | -94.87 |
| Conf G | 5.14 | 80.73 | 55.68 | 64.12 | 75.91 |
| Conf H | 5.00 | -70.29 | -62.94 | -59.72 | -69.90 |
| Conf I | 4.65 | 148.52 | 119.58 | 128.45 | 140.59 |
| Conf J | 3.69 | 58.00 | 36.77 | 45.74 | 53.61 |
| Conf K | 3.13 | -45.18 | -36.87 | -36.70 | -43.87 |
| Conf L | 2.64 | -68.47 | -72.58 | -67.38 | -69.01 |
| Conf M | 2.45 | -50.99 | -41.29 | -40.31 | -50.26 |
| Conf N | 2.29 | 55.13 | 38.35 | 44.27 | 48.32 |
| Conf O | 2.11 | -73.24 | -73.82 | -69.30 | -74.16 |
| Conf P | 1.96 | 56.03 | 41.84 | 45.07 | 52.16 |
| Conf Q | 1.82 | 35.56 | 22.30 | 28.48 | 28.61 |
| Conf R | 1.75 | -176.10 | -151.82 | -155.11 | -172.35 |
| Conf S | 1.19 | 22.78 | 2.98 | 12.94 | 10.38 |
| Conf T | 1.16 | -33.80 | -41.39 | -38.43 | -38.72 |
| Conf U | 1.15 | -30.50 | -22.32 | -17.68 | -35.14 |
| Conf V | 1.06 | -14.38 | -4.92 | -5.29 | -15.06 |
| Conf W | 1.05 | -103.87 | -68.95 | -73.87 | -101.42 |
| Conf X | 1.05 | 5.62 | 5.71 | 6.32 | 3.19 |
| Conf Y | 1.01 | -8.01 | -5.83 | -5.93 | -9.62 |
| Conf Z | 0.99 | 107.71 | 85.88 | 94.45 | 92.45 |
| BW Average |  | -20.08 | -22.44 | -18.19 | -22.56 |
